# Mild Focal Cooling Decouples Dendrites to Reconfigure Cortical Output

**DOI:** 10.1002/advs.202520773

**Published:** 2026-04-09

**Authors:** Meisam Habibi Matin, Shulan Xiao, Krishna Jayant

**Affiliations:** ^1^ Weldon School of Biomedical Engineering Purdue University West Lafayette Indiana USA; ^2^ Purdue Institute for Integrative Neuroscience Purdue University West Lafayette Indiana USA

**Keywords:** dendritic excitability, differential sensitivity, focal cooling, neuromodulation, physiology

## Abstract

Focal cooling modulates cortical computations, yet how principal neurons respond remains unclear. We demonstrate that mild focal cooling of the barrel cortex (S1) while impacting behavior creates a steep translaminar temperature gradient with ∼4°C drop in layer 5 (L5)—a range where conduction velocity changes are minimal. L5 neurons integrate self‐motion and touch by encoding whisker dynamics, and because their apical tuft dendrites lie proximal to the cooled surface, the gradient implicates possible dendritic mechanisms in mediating behavioral disruption. In vitro experiments confirm this: focal cooling (100 µm radius) selectively increases impedance and input‐output transformations in L5 tuft but not basal dendrites, yet paradoxically impairs recovery from inactivation of apical dendritic Na^+^ channels, reducing somato‐dendritic coupling. These results challenge the view that cooling acts mainly through slowed conduction. Instead, suggesting that cooling decouples basal‐tuft integration and dynamically regulates cortical gain, revealing a potent neuromodulatory mechanism with implications for sensory‐motor computation.

## Graphical abstract

Mild cooling of the cortical surface selectively modulates apical dendritic excitability, plasticity, and somato‐dendritic coupling, while uncoupling these effects from basal dendrites, and reshapes apical‐driven responses in barrel cortex during whisker touch.

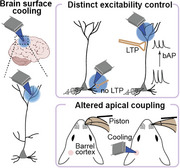

## 1 Introduction

Compared to neuromodulation techniques such as optogenetics and chemogenetics, which digitally control neural circuit activity by turning it on or off [1, 2], mild focal cooling of the brain allows for graded (analog) changes in temporal dynamics [3, 4, 5]. This temporal scaling has been found to influence behavior and reveal the circuit functions involved in timing‐dependent computations [5, 6, 7]. Notably, cooling from the brain's surface has been shown to change the temporal pattern of neural activity, inducing changes in behavior, without disrupting the intrinsic structure [4, 8], an effect often attributed to reduced axonal conduction speed, i.e., “slowing down activity”. However, in the cortex, this axon‐centric view neglects another feature uniquely sensitive to surface cooling — the apical dendrites of large layer 5 (L5) pyramidal neurons, which extend from deep output layers to the superficial layers directly under the cooled surface. These dendrites could be critical in mediating temperature‐dependent changes in circuit dynamics—especially given their crucial role in integrating synaptic inputs and shaping output in cortical neurons.

L5 neurons integrate long‐range feedback in apical tufts, while local and feedforward inputs converge on their basal dendrites [9, 10, 11], forming computations which are paramount for cortical information processing [11, 12, 13]. The tufted dendrites in the upper cortical layers and basal dendrites in the deeper cortical layers are compartmentalized for different types of integration [14, 15, 16, 17, 18]. The tufted dendrites, located in the upper cortical layers, primarily integrate inputs from higher cortical areas, as well as top‐down influences that affect cognitive functions such as attention and perception [9, 19]. In contrast, the basal dendrites, found in the deeper layers of the cortex, mainly process local cortical and thalamic inputs, contributing to bottom‐up information processing [11, 20, 21]. This extensive dendritic arborization of L5 neurons creates a unique structure where temperature gradients, applied from the brain surface, can differentially affect distinct integration zones within the same neuron—a specialization less pronounced in more superficial neurons such as those in layers 2/3. This distinction is significant because simple changes in conduction speed alone cannot explain how cortical cooling can eliminate silent network states [22]. Benchmarking how temperature affects these different dendritic regions from an integration standpoint can reveal how thermal changes might selectively influence feedforward and feedback synaptic streams [23, 24, 25, 26], a feature not readily possible with optogenetics. Overall, this underscores the importance of investigating dendritic‐domain‐specific effects of mild cooling in L5 neurons, given their critical role in modulating network states, cortical output, and behavior.

Computational models and previous studies have shown that lowering temperature can affect fundamental characteristics of neuronal membranes, such as their ability to modulate excitability [27, 28, 29, 30], reduce membrane fluidity [31, 32], influence metabolism [33], impact impulse propagation [28, 34, 35, 36, 37, 38, 39], and regulate synaptic transmission [40, 41, 42, 43, 44]. However, how selective/focal temperature changes affect neuronal computations remains unclear, especially across different neural compartments. Such focal reductions in temperature effects could locally modulate the temperature dependence of voltage‐gated ion channel activity, influence excitability through impedance changes, and ultimately impact dendritic computation in a compartmentalized manner—a feature we uncover in L5 neurons.

Temperature can also significantly impact excitability via synaptic plasticity mechanisms like long‐term potentiation (LTP) and long‐term depression (LTD) [45, 46], which are crucial for learning and memory [47]. Since tuft and basal dendrites have distinct synaptic and signaling pathways [13, 48, 49, 50, 51, 52], temperature changes could affect these processes differently, for example, via changes in calcium dynamics [53] or local excitability [54]. Understanding how temperature influences plasticity and excitability in turn in these distinct dendrites is thus critical. Additionally, the reversible nature of focal cooling would also allow us to observe how plasticity processes recover after the cooling is removed. These recordings can provide insights into the adaptability and thermal resilience of neural circuits [55], a feature critical for optimal engineering of cooling as a neuromodulation technique.

The differential impacts of cooling across a neuron are particularly critical, as previous studies are at odds with one another. Notably, some reports have indicated that neuronal excitability increases in the temperature range of 15°C–20°C before being suppressed around 10°C [28, 43]. Conversely, some studies show a net slowing of spike output as a function of temperature, although within a moderate fluctuation range (ΔT <10°C) [56]. Thus, not all neuronal locations respond to temperature similarly, so the net output as a function of temperature represents a unique integration of these mechanisms. There is thus a need in cooling‐based neuromodulation to systematically characterize, model, and validate the membrane, cellular, and adaptive‐biological responses.

In this study, we demonstrate that focal cooling of S1, at levels sufficient to induce behavior change, creates a steep translaminar gradient, selectively increasing impedance and altering the input‐output relationships in L5 tuft dendrites, while impairing apical Na^+^ channel recovery. These reversible, location‐specific effects demonstrate that cortical cooling alters integration in a compartment‐specific fashion, not uniformly across neurons. This finding challenges the prevailing axonal conduction‐only hypothesis and reframes how focal cooling should be interpreted in behavioral and circuit studies.

## 2 Results

### 2.1 Focal Cortical Cooling Modulates Behavior while Inducing a Temperature Gradient

**FIGURE 1:**
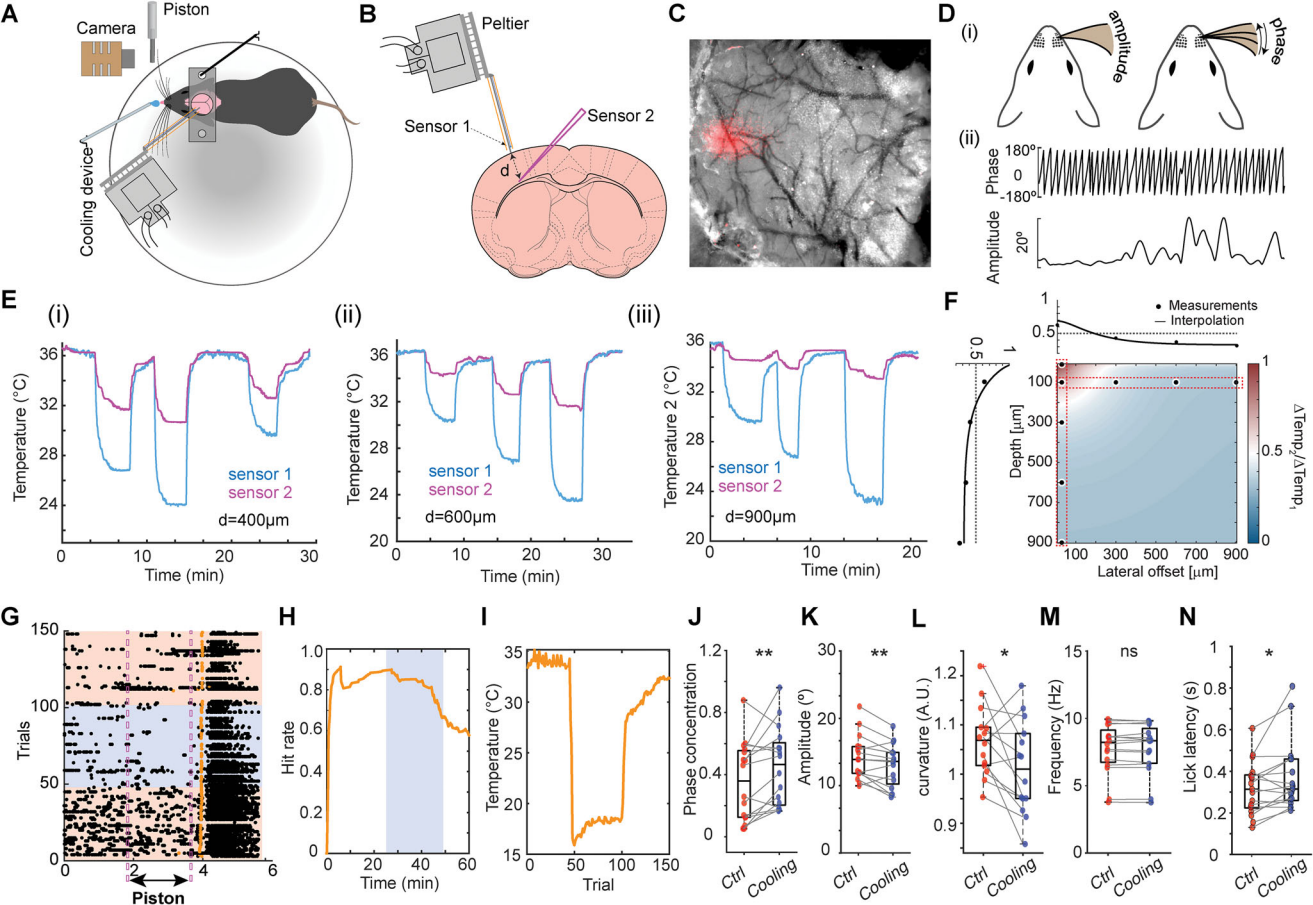
Focal cortical cooling modulates behavior while inducing a temperature gradient (A) Schematic of the experimental setup showing the Peltier cooling device positioned over the barrel cortex (S1) of a head‐fixed mouse locomoting on a treadmill and actively whisking. Dual thermocouple sensors are inserted to monitor superficial (sensor 1) and deeper (sensor 2) cortical temperatures, and a high‐speed camera and digital encoder recorded whisker position and locomotion speed, respectively. (B) Diagram illustrating placement of the Peltier element and sensors relative to the mouse cortex in a coronal view. (C) Intrinsic imaging confirming the C1 barrel targeted for cooling. (D) (i) Illustration of slow‐varying whisker features—amplitude (left)—and the fast‐varying feature, whisker phase (right). (ii) Example trial showing time‐course of whisker phase (top), and amplitude (middle). (E) Representative temperature traces at cortical depths of 400 µm (i), 600 µm (ii), and 900 µm (iii). Surface cooling (sensor 1, cyan) produced a robust reduction in temperature (∼12°C), whereas deeper layers (sensor 2, magenta) experienced a smaller drop (∼3°C), establishing a stable vertical temperature gradient. (F) A 2D map fitted with experimental recordings in A), the black dots show the experimentally measured locations. (G) Raster plot showing trial‐wise lick in response to sensory stimulation during control (red) and cooling (blue) epochs. (H) Hit rate over the session (blue epoch corresponds to the cooling period). (I) Cortical temperature across trials. (J–N) Summary of behavioral metrics across 16 sessions in 4 mice. Cooling significantly increased whisking phase concentration (I, *p* < 0.01), decreased whisker amplitude (J, *p* < 0.01) and curvature (K, *p* < 0.05), did not have significant effect on whisking frequency (L, ns), and delayed lick latency (M, *p* < 0.05), suggesting that superficial cortical cooling disrupts fine‐scale sensorimotor processing. Each line represents one session; red and blue dots represent control and cooling conditions, respectively. Linear mixed‐effects model is used to test the statistical significance.

We asked the following question: What is the nature of the temperature profile during mild focal cooling from the cortical surface, and how does this profile influence cortical output? To investigate this, we explored how focal cortical cooling shapes in vivo temperature distributions and, in turn, impacts active touch behavior. Experiments were conducted in complete darkness to eliminate visual cues, and mice performed a whisker‐based detection task using only their intact C1 whisker to sample a piston, earning water reward upon correct responses (Figure 1A, see Methods). A thermoelectric Peltier‐driven cooling probe (Figure 1A,B;  Figure S1A–C) was positioned with high spatial precision (Figure S2) over the C1 barrel, identified by intrinsic signal imaging (Figure 1C), enabling localized cortical cooling. Head‐fixed mice, free to whisk and locomote on a treadmill [57, 58, 59, 60, 61, 62, 63], were monitored with high‐speed infrared imaging (250 fps), and whisker movements were quantified using markerless tracking [64], providing precise measurements of phase, amplitude, and frequency (Figure 1D, see Methods). Temperature was measured simultaneously at the probe surface and at cortical depths of 400, 600, and 900 µm (Figure 1B, see Methods) to map translaminar cooling gradients. Across three progressively stronger cooling conditions, the cortical surface temperature fell by approximately 14°C, yet temperature decreases at 600 and 900 µm depths remained modest, below 4°C and 3°C, respectively (Figure 1E,F). This established a steep translaminar gradient, suggesting that axon and proximal dendrites of L5 pyramidal neurons (600–900 µm depth) [65, 66, 67, 68] experience only a modest temperature change by ∼3°C–4°C. We then assessed behavioral consequences of this cooling during touch processing. Raster plots of trial activity revealed consistent performance across cooling conditions (Figure 1G,H), indicating that mice remained engaged. Cooling nonetheless induced a rapid and reproducible drop in cortical temperature (Figure 1I), which significantly altered whisking dynamics: whisker phase concentration increased, whereas whisker amplitude and curvature decreased, reflecting disrupted coordination (Figure 1J–L, n = 4 animals, 16 sessions; p = 0.009 for concentration, p = 0.005 for amplitude, linear mixed‐effects test). Whisking frequency, however, remained unchanged (Figure 1M, p > 0.05), indicating that rhythmicity was preserved, but the structure and precision of whisk movements were impaired.

Because changes in whisker kinematics could reflect altered motor coordination rather than impaired tactile perception, we performed additional analyses to assess the perceptual relevance of cooling‐induced behavioral effects. First, we examined whisker movements during hit‐and‐miss trials in the whisker‐based detection task under control conditions. Successful detection (hit trials) was associated with more precise whisker positioning around the stimulus location and with larger contact‐induced curvature than in miss trials, indicating that accurate whisker sampling correlates with perceptual outcome (Figure S3). Although correlative, this analysis establishes a relationship between whisker‐kinematic precision and tactile‐detection performance.

To more directly probe tactile sensitivity, we next employed a whisker‐based GO/NOGO discrimination task with higher cognitive demand. In this task, animals were required to distinguish tactile inputs delivered to adjacent whiskers (C1 vs. D1). Mild focal cooling significantly reduced discrimination sensitivity (d′), an effect that reversed upon bringing temperatures back to baseline (Figure S4A–C). Consistent with the detection task, cooling also disrupted the spatial precision of whisker positioning relative to the stimulus during discrimination trials, despite comparable locomotor behavior (Figure S4D,E). Notably, cooling also led to a small yet significant increase in lick latency following whisker‐piston contact (Figure 1N, n = 4 animals, 16 sessions, p = 0.036), suggesting a delay in goal‐directed tactile processing. Interestingly, focal cooling also reduced the spontaneous licking behavior prior to the presentation of piston stimulation (Figure S5), suggesting that the behavioral effect could reflect a combination of altered sensorimotor processing and overall response strategy emanating from S1. From a mechanistic viewpoint, our results suggest that the disruption of goal‐directed behaviors by localized cortical cooling has a locus in superficial layers, for example through modulations of dendritic activity in L5 pyramidal neurons, since infragranular somata experienced only modest temperature shifts of 3°C–4°C.

### 2.2 Focal Cooling Differentially Modulates L5 Dendritic Excitability

**FIGURE 2:**
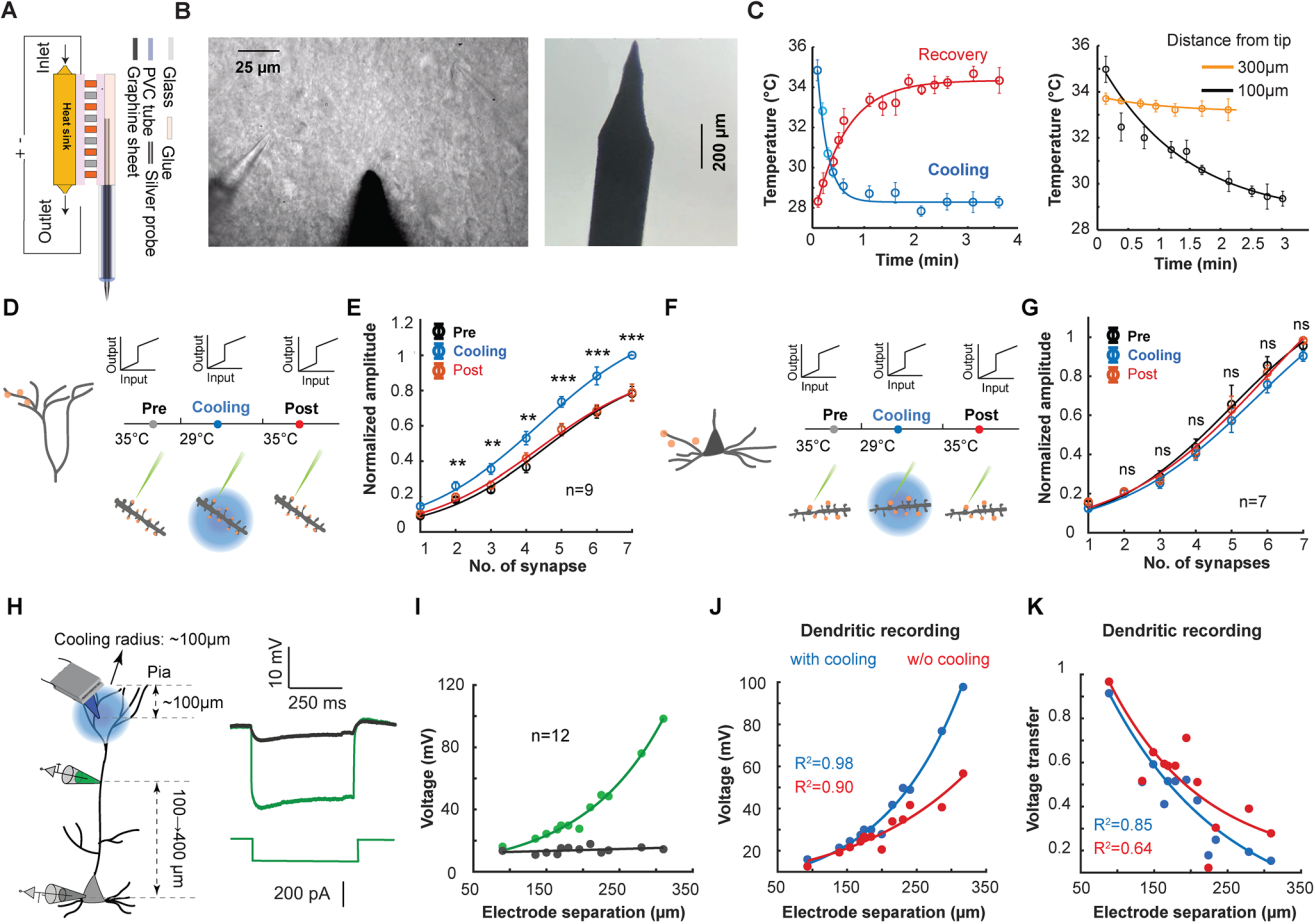
Focal cooling differentially modulates L5 dendritic excitability (A) Schematic of the focal cooling device (B) Double contrast of the recording and stimulation pipettes, and the cooling probe on the slice (left), and the tip of the sharpened cooling probe under the microscope. (C) Temperature measured for cooling/recovery experiments (left), and at different distances from the tip of the cooling probe (right). Note: At a distance of ∼300 µm from where the cooling probe tip is, the drop in cooling is not significant, reflecting a ∼ 0.5°C drop in the temperature; however, at a 100 µm distance from the probe tip, the temperature drop is substantial (5°C–6°C). Error bars represent the standard error of the mean (SEM). (D) Schematic of the experimental protocol for probing the temperature dependence of excitability via a readout of input‐output transformations across the tuft dendrite. Two‐photon glutamate uncaging probes input‐output characteristics before (control, gray), during (blue), and after (post, red) cooling. (E) Normalized dendritic input‐output response measured at the tuft dendrites before (35^°^C), during (29^°^C), and after (35^°^C) focal cooling. Error bars represent the standard error of the mean (SEM). (F,G) Uncaging experiments as shown in (D,E), but for the basal dendrites. (H) Schematic of the dual somatic and apical dendritic patch clamp recording while cooling the tuft dendrite (left). The voltage response from a subthreshold current injected in the dendrite (green) and the soma (gray). (I) Measurement of the dendro‐somatic (dendritic injection) voltage responses at steady state under cooling. (J) Comparison between voltage attenuation along the dendro‐somatic (dendritic injection) axis under baseline (red) and under mild focal cooling at the tuft (blue). Recordings are from the apical dendrite and solid lines represent exponential fits to the data. Voltage attenuation increases by cooling closer to the tuft dendrite (n = 12 neurons, *p* = 0.017, Repeated‐Measures ANOVA). (K) Voltage transfer, the ratio of voltage recorded at the soma to the voltage recorded at the dendrite, is lower in the condition with mild focal cooling present across the tuft (n = 12 neurons, *p* = 0.001, Repeated‐Measures ANOVA).

Given the measured gradient in temperature along the cortical axis, we hypothesized that cortical output might be impacted by the impact of cooling on dendritic excitability. To test this theory, we performed ex vivo electrophysiological recordings from both somata and dendrites of L5 pyramidal neurons from the primary somatosensory cortex of the mouse (see Methods) while focally cooling specific dendritic segments using our custom‐designed focal cooling probe (Figure 2A,B). This ensured a spatial spread of temperature in the slice similar to that observed in vivo (∼100–150 µm) (Figure 2C). It is critical to note that although the thermistor in the bath was set to 37°C, due to perfusion and flow, the measured temperature was observed to be 35°C. Under this condition, the probe tip was cooled by 5°C–6°C to reach ∼29°C–30°C. We further corroborated probe efficiency through thermal modeling (Figure S6A,B).

To probe dendritic excitability changes, we first employed two‐photon glutamate uncaging across both basal and tuft dendrites (see Methods) to map input‐output characteristics before (control, gray), during (blue), and after (post, red) moderate cooling for each of these compartments (Figure 2D,G). Uncaging a sequence of 1–7 closely spaced dendritic spines (distributed along 10–20 µm stretches of a single branch) resulted in a sigmoidal input‐output transformation measured at the soma, consistent with previous studies [18, 26, 69, 70]. The results revealed that during focal cooling, the input‐output transformations in the tuft dendrites were enhanced. This enhancement was observed to be reversible upon establishment of the baseline physiological temperature (Figure 2E, n = 9 neurons, p < 0.001 for 5–7 spines and p < 0.01 for 2–4 spines, Repeated‐Measures ANOVA test), indicating that temperature serves as an analog modulator without any long‐term changes to dendritic input‐output transformations. Under the same conditions, but with mild focal cooling applied across basal dendrites, the basal dendritic input‐output transform remained largely unaffected (Figure 2F,G, n = 7 neurons, ns, p > 0.05, Repeated‐Measures ANOVA test).

The reversible effect of cooling on the excitability was also supported by the field‐evoked EPSP measurement (see Methods) before, during, and after cooling (Figure S7A,B, n = 8 neurons, w cooling: p = 0.0014, w/o cooling: p > 0.05, Repeated‐Measures ANOVA test). Since voltage‐gated potassium channels are critical for membrane excitability, and given the high temperature sensitivity of A‐type potassium channels [37], particularly Kv4.2, their enriched expression in tuft dendrites [71, 72, 73], and their involvement in branch‐specific excitability [74], we posited that these channels are critical for the cooling‐induced enhancement in dendritic excitability observed in the tuft arbors. To test this, we applied 400 nm heteropodatoxin‐2, a selective Kv4.2 channel blocker, to the bath solution. After a 20‐min incubation period, we repeated the two‐photon glutamate uncaging along tuft branches to probe the input‐output characteristics and compared the response to conditions before and after cooling. Under Kv4.2 blockade, the uncaging‐evoked EPSPs recorded at the soma no longer exhibited the cooling‐induced enhancement in excitability or input‐output transformation, supporting the role of Kv4.2 channels in mediating the cooling‐induced enhancement (Figure S7C,D, ns, p > 0.05, Repeated‐Measures ANOVA test).

We next investigated whether the impact of cooling across tuft arbors modulates electrical compartmentalization along the apical dendrite. To test this, we performed dual whole‐cell recordings from the soma and apical dendrite of L5 pyramidal neurons while focally cooling the apical tuft (Figure 2H–K). By injecting subthreshold current pulses at either the soma or the dendrite, we measured the voltage at both locations. This allowed us to assay how mild cooling affects the membrane resistance and resultant voltage attenuation profile along the somato‐dendritic axis [75]. Under mild cooling across the tuft (∼5°C–6°C), we observed an increase in voltage attenuation along the dendro‐somatic axis (Figure 2J, n = 12 neurons, p = 0.017, Repeated‐Measures ANOVA; Figure 2K, p = 0.001, Repeated‐Measures ANOVA). These changes were not observable when measured from the soma (Figure S8A,B), suggesting a localized change in the apical dendrites. Physiologically, these results suggest that cooling enhances dendritic excitability via elevated input resistance, which in turn results in more dampened voltage propagation.

### 2.3 Tuft Dendritic Cooling Impacts Somato‐Dendritic Coupling

Back‐propagating action potentials (bAPs) can critically impact neural dynamics, including burst firing and plasticity. We posited that the change in voltage transfer observed along the apical direction due to tuft cooling (Figure 2) could modulate the nature of bAP propagation and, in turn, burst firing.

**FIGURE 3:**
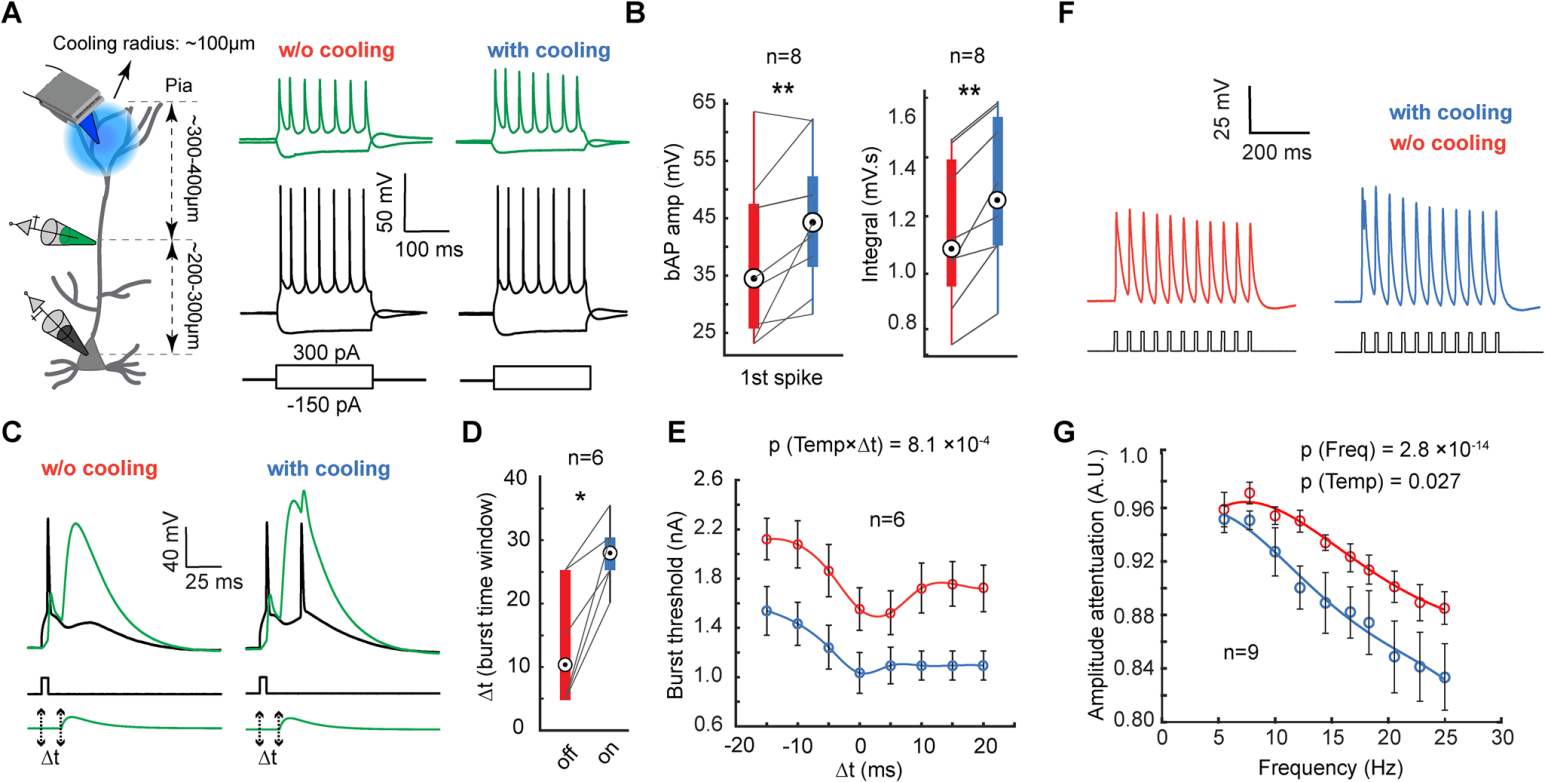
Focal cooling of the tuft impacts somato‐dendritic coupling (A) Schematic of the dual somatic and apical dendritic patch clamp recording while cooling the tuft dendrite (left). Prototypical spike trains recorded at the soma (bottom) and apical dendrite (top) under moderate focal cooling of the tuft dendrite at T = 29°C (right) and T = 35°C (left). (B) The amplitude comparison of the first bAP spike (left) (Wilcoxon signed rank test, n = 8 neurons, *p* = 0.0078), and the integral (right) (Wilcoxon signed rank test, n = 8 neurons, *p* = 0.0039) comparing conditions with and without mild focal cooling. (C) The combination of current injection at the soma with EPSP‐ like depolarization in the apical dendrite separated by a time interval (Δt) of 10 ms evoked a burst following the onset of Ca^2+^ ‐AP in the apical dendrite. (left) when the tuft dendrite is not cooled and (right) with mild focal cooling. (D) The time duration (Δt) needed to generate the burst is compared for cooled (on) and control conditions (off). The time window for coincidence detection and burst generation increases by cooling. (E) Burst threshold as a function of the relative timing (Δt) between somatic and dendritic inputs under control (red) and focal cooling (blue) conditions. A linear mixed‐effects model with ANOVA‐style F‐tests revealed significant main effects of temperature (*p* = 1.3 × 10^−^^4^) and Δt (*p* = 4.8 × 10^−^^7^), as well as a significant Temperature × Δt interaction (*p* = 8.1 × 10^−^^4^), indicating that focal cooling alters the temporal dependence of burst generation. Error bars represent the standard error of the mean (SEM). (F) Train of action potential generated at the apical dendrite to measure the spike amplitude attenuation under cooling and control conditions. (G) Frequency‐dependent attenuation of back‐propagating action potential (bAP) amplitude under baseline and focal cooling conditions. Attenuation was quantified as the ratio of the amplitude of the last spike in an eleven‐spike train to that of the third spike. A two‐way repeated‐measures ANOVA analysis revealed a significant main effect of temperature (*p* = 0.0268) and spike frequency (*p* < 10^−^^14^), while the temperature × frequency interaction was not significant (*p* = 0.32), indicating that focal cooling induces a largely frequency‐independent increase in bAP attenuation. Error bars represent the standard error of the mean (SEM).

To explore this facet, we conducted whole‐cell somato‐dendritic patch‐clamp recordings to map coupling between the soma and apical dendrite (200–350 µm from the soma) under the influence of mild focal cooling applied across the tuft (Figure 3A). We induced bAPs via somatic current injection, mapped the amplitude at both at the soma and dendrite under tuft cooling (at 29°C), and compared the responsivity to recordings performed under physiological baseline (35°C). We observed changes in both the amplitude and the integral area under the curve as a function of cooling (Figure 3B). Specifically, we observed a significant increase in the first bAP spike amplitude from 35.5±3.86 mV at 35°C to 43±3.85 mV under cooling, reflecting a 25% average increase (Figure 3B, n = 8 neurons, p = 0.008, Wilcoxon signed‐rank test). The integral area under the curve increased from 1.1 mV·s at 35°C to 1.3 mV·s at 29°C (n = 8 neurons, p = 0.008, Wilcoxon signed‐rank test).

We next assayed if such a change leads to shifts in burst firing mediated by somato‐dendritic coupling, as evidenced via back‐propagating action potential‐activated Ca^2+^ spike firing (BAC firing) (Figure 3C–E). Here, combining a subthreshold EPSP‐shaped distal dendritic potential and a bAP within a time duration Δt elicited a complex Ca^2+^ spike in the dendrite. We observed an apparent increase in BAC firing efficiency as a function of tuft cooling. Namely, the time window (Δt) for burst induction was more prolonged (Figure 3D, 13.5 ± 4.01 ms without cooling compared to 27.5 ± 2.14 ms under cooling; n = 6 neurons), while at the same time the threshold to generate the burst was reduced (Figure 3E). A linear mixed‐effects analysis revealed significant main effects of temperature and Δt, as well as a significant Temperature × Δt interaction (p = 8.1 × 10^−^^4^), indicating that focal cooling alters the temporal dependence of burst generation. Strikingly, however, bAP amplitude attenuated more severely as a function of spike frequency under moderate focal cooling (Figure 3F,G). We assayed this attenuation effect as follows. We measured the ratio of the bAP amplitude of the last spike (in a train of eleven spikes) to that of the third spike. Our results indicate that bAP attenuation was more pronounced at 29°C and increased with higher bAP frequencies (Figure 3G). A two‐way repeated‐measures analysis revealed a significant main effect of temperature (p = 0.0268) and a strong main effect of spike frequency (p < 10^−^^14^), indicating that focal cooling induces a robust increase in bAP attenuation across frequencies. The temperature × frequency interaction was not significant (p = 0.32), suggesting that cooling produces a largely frequency‐independent shift in attenuation rather than selectively altering its frequency dependence. This increased attenuation under cooling is most likely due to prolonged recovery from the inactivation of voltage‐gated sodium channels as a function of temperature, which reduces bAP spike amplitude [76, 77].

The results presented in Figures 2 and 3 suggest a paradox: mild focal cooling depolarizes the tuft and increases excitability, yet it simultaneously reduces the efficacy of frequency‐dependent back‐propagating action potential (bAP) invasion, thereby controlling bursting. Notably, while the apical dendritic impedance increases with tuft cooling, the diminished recovery from inactivation of Na^+^ channels limits the efficiency of coupling. To assess whether selective modulation of tuft excitability is sufficient to influence cortical output in vivo, we performed optogenetic activation or inhibition of distal apical dendrites. Optogenetic modulation produced changes in sensory‐evoked cortical responses that were directionally consistent with the effects observed under focal cooling (Figure S9). These results provide complementary causal support for the role of tuft dendritic excitability in shaping cortical processing.

### 2.4 Focal Cooling Modulates Tuft but Not Basal Dendritic Plasticity

**FIGURE 4:**
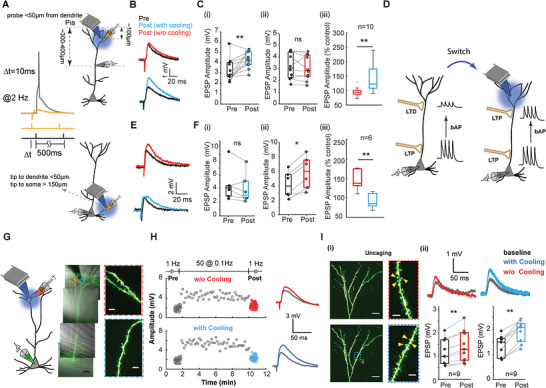
Focal cooling enhances the synaptic potentiation on apical but not basal dendrites (A) A spike timing‐dependent plasticity (STDP) induction used to evoke potentiation across the tuft dendrite induction in the presence and absence of mild focal temperature modulation. Baseline (black) and post‐induction readout without cooling (red) or with cooling (blue) applied. (B) Example representative EPSPs for baseline temperature (35°C, black), and post‐induction readout without cooling (red) and with cooling (blue) applied. (C) Comparison of evoked amplitudes before and after STDP without cooling (left), with cooling (middle), and comparison of percentage changes in EPSP amplitude for the two cases with and without cooling. A mild reduction in temperature during plasticity induction enhances the STDP‐induced plasticity. (D) Cooling‐induced dendritic excitability biases STDP toward potentiation in tuft by boosting backpropagation of action potential. (E‐F) The same results as in (B,C) obtained for the basal dendrite. (G) Schematic depicting low‐frequency stimulation‐induced plasticity in basal dendrites under mild focal cooling (left). The green pipette represents the somatic whole‐cell patch pipette used for dual somato‐dendritic recordings, and the orange pipette represents the extracellular stimulating electrode used to evoke synaptic responses in the dendrites. An exemplar layer 5 pyramidal neuron loaded with Alexa 488 (100 µm) (scale bars, 50 µm). (Inset) dendritic segments that underwent the plasticity protocol without (top, red) and under the influence of focal cooling (bottom, blue) (scale bars, 5 µm). Note: distance from the tip of the cooling probe to the targeted dendrite is ∼50 µm. (H) Field‐evoked EPSP amplitude as a function of low‐frequency plasticity induction in the presence and absence of mild focal temperature modulation. The initial readout at 1 Hz (gray) is followed by potentiation at 0.1 Hz and subsequent readout again at 1 Hz (blue). While the initial (gray) and final readouts (blue) are performed at 35^°^C, the plasticity induction is carried out at two different temperatures for comparison – 35^°^C (data not shown) and 29^°^C (gray). For clarity, the initial and final readouts at 1 Hz, as well as the potentiation (0.1 Hz) are only shown for the case in which plasticity was induced at 29^°^C. Note: the induced plasticity is amplified under the influence of mild focal cooling (post vs pre with cooling: 153%±11%, *p* = 0.0039; post vs. pre w/o cooling: 122%±5.6%, *p* = 0.0068; Wilcoxon signed‐rank test). (I) (i) Uncaging‐evoked responses from spines located on tuft dendritic branches that underwent plasticity induction in the absence of cooling (top, red) and under the influence of cooling (bottom, blue) (scale bars, 50 µm). (Inset) Dendritic segments where the spines are uncaged before and after plasticity induction without (top, red) and under the influence of focal cooling (bottom, blue) (scale bars, 5 µm). (ii) The induced plasticity is amplified under the influence of mild focal cooling (with cooling: 157%±13%, n = 9 neurons, *p* = 0.0039; w/o cooling: 131.5%±10%, n = 9 neurons, *p* = 0.0039; Wilcoxon signed‐rank test). Error bars represent the standard error of the mean (SEM).

Since membrane excitability, specifically depolarization, and bAP efficiency are critical for plasticity, we next asked whether the focal cooling‐induced bAP changes observed affect the nature of plasticity, both across the tuft and basal arbors. To test this, we examined spike‐timing‐dependent synaptic plasticity (STDP) using established timing protocols [78, 79] (see Methods) under mild focal cooling and at physiological temperature (Figure 4A). In tuft dendrites, the application of mild cooling during STDP induction resulted in a significant increase in post‐induction EPSP amplitude (Figure 4B,C (i), n = 10 neurons, p = 0.007, Wilcoxon signed‐rank test). In contrast, the same protocol applied at physiological baseline temperatures produced no significant change (Figure 4B,C (ii), n = 10 neurons, p = 0.232, Wilcoxon signed‐rank test). To quantify inter‐neuronal variability and uncertainty given the sample size, we additionally examined the distribution of plasticity magnitude (ΔEPSP = EPSP_post_ − EPSP_pre_) across individual neurons and report the mean ± 95% confidence interval (Figure S10). It is critical to note that the bAP applied here is a dendritically‐evoked spike and not a somatic current injection‐induced spike. Indeed, somatically evoked single spikes almost fail to invade the distal tuft and generate a regenerative potential, in line with previous reports [80, 81].

Strikingly, in contrast, STDP induction across basal dendrites at 29°C failed to produce potentiation under focal cooling (Figure 4E,F (i); 87 ± 7.2% of control; Wilcoxon signed‐rank test, n  =  6 neurons, p  = 0.156), whereas the same protocol at the baseline temperature led to 44% increase in EPSP amplitude (Figure 4E,F (ii); 144 ± 17% of control; n = 6 neurons, p = 0.031, Wilcoxon singed‐rank test). Indeed, the change in EPSP amplitude before and after STDP induction showed a significant depression under mild focal cooling compared to physiological temperature conditions (Figure 4F (iii); p = 0.021, Repeated‐Measures ANOVA). Here, the bAP was somatically evoked. The above compartment‐specific shift in excitability suggests that mild focal cooling, via changes in passive membrane impedance, increases bAP invasion into the distal dendrites, consistent with models where changes in excitability can alter the effective sign of plasticity (Figure 4D) [49]. A soma‐generated burst of three action potentials elicited a higher calcium electrogenesis in the tuft dendrite under mild focal cooling compared to baseline conditions (Figure S11A,B, p = 0.002, One‐way ANOVA). This, however, was not the case across proximal dendrites, suggesting differential modulation of dendritic dynamics by temperature (Figure S11B).

We next asked whether temperature and location influence synaptic efficacy in ways distinct from those seen with high‐frequency or STDP‐based induction, making it essential to determine if cooling‐driven improvements in backpropagating action potential invasion and synaptic plasticity persist under more physiologically relevant patterns of neural activity. To this effect, we performed low‐frequency plasticity induction experiments across the tuft and basal dendrites (Figure 4G–I; Figure S12A–F) using a novel form of N‐methyl‐D‐aspartate receptors (NMDARs) and Kv4.2‐dependent potentiation [50]. We stimulated tuft dendrites with low‐frequency (0.1 Hz) single unpaired EPSPs (see Methods) to induce plasticity [50] (Figure 4G,H). This protocol was performed both at the physiological baseline (35°C) and under mild focal cooling (Figure 4H). The pre‐stimulation baseline and final post‐plasticity‐induction readouts were performed at the physiological baseline temperature of 35^°^C under a 1 Hz stimulation (Figure 4H), revealing a net increase in EPSP amplitude when plasticity was induced under mild focal cooling (Figure 4H and Figure S12C, post vs. pre with cooling: 153%±11%, n = 9, p = 0.0039; post vs. pre w/o cooling: 122%±5.6%, n = 10, p = 0.0068; Wilcoxon signed‐rank test). This effect was absent in basal dendrites, revealing instead a decrease in the EPSP amplitude regardless of the temperature (Figure S12F, n = 9 neurons, p = 0.012 (w/o cooling); n = 10, p = 0.015 (with cooling); within‐condition comparisons: Wilcoxon signed‐rank test). Notably, the resultant EPSP after induction of tuft dendritic plasticity persisted long after focal cooling was removed (Figure S13A,B: Wilcoxon Signed‐rank test, n = 6 neurons, ^*^p <.05). Direct comparison of the time course between cooling and no‐cooling conditions revealed a significant Group × Time effect (Figure S13D; mixed Group × Time ANOVA implemented as a linear mixed‐effects model, p = 9.8 × 10^−^^5^). This cooling‐induced effect in tuft dendrites was most evident in the input–output relationship, where identical synaptic inputs produced larger dendritic responses when plasticity was induced under cooling compared to the control condition (Figure S14A–D).

We then validated that the EPSP increase was indeed post‐synaptic by performing pre‐ and post‐induction readout using two‐photon glutamate uncaging (∼3 nearby spines; see Methods for details) located along tuft dendritic branches (Figure 4I (i)). It is critical to note that the plasticity induction and recordings were done under bath application of Gabazine (Figure S15, and see Methods). This ensured a stable EPSP amplitude to compare the effect of mild focal cooling on plasticity. The post‐induction readout was measured at least 10 min after the low‐frequency induction to ensure that the potentiation effect is not transient. It should be noted that a full recovery from 29°C to 35°C spans 3–4 min (see Figure 1), and the post‐LTP recording was carried out after a 10‐min settling‐down period. Dendrites that underwent plasticity under mild focal cooling exhibited a 157%±13% increase in the EPSP amplitude (Wilcoxon signed‐rank test, n = 9 neurons, p = 0.0039) after low‐frequency plasticity induction. In contrast, dendrites that underwent plasticity at the physiological baseline temperature reflected a 131.5%±10% increase in EPSP amplitude (Wilcoxon signed‐rank test, n = 9 neurons, p = 0.0039) (Figure 4I (ii)). These uncaging‐evoked responses also revealed increases in spine calcium (see Methods) when plasticity was induced under mild cooling compared to the induction at physiological temperature (Figure S16, n = 5 neurons, p = 0.04, Repeated‐Measures ANOVA). We found that the Kv4.2 potassium channel, previously identified as critical for cooling‐induced excitability modulation in tuft dendrites (Figure 2), is also essential for cooling‐enhanced plasticity in these compartments (Figures S17 and S18). This aligns with the well‐documented high temperature sensitivity of A‐type potassium channels [37], particularly Kv4.2, and with their established roles in low‐frequency‐induced long‐term potentiation (LTP) [50], other forms of LTP [82], and branch‐specific excitability within tuft dendrites [74].

The findings in Figures 3 and 4 demonstrate that mild focal cooling not only enhances bAP invasion and selectively potentiates synapses in tuft dendrites, but also fundamentally shifts the direction and magnitude of synaptic plasticity in a branch‐specific and temperature‐dependent manner—revealing a powerful modulatory mechanism through which local temperature dynamics can shift dendritic excitability and plasticity thresholds, with lasting consequences for circuit function.

### 2.5 Focal Cooling of the Tuft Modulates Neuronal Gain

**FIGURE 5:**
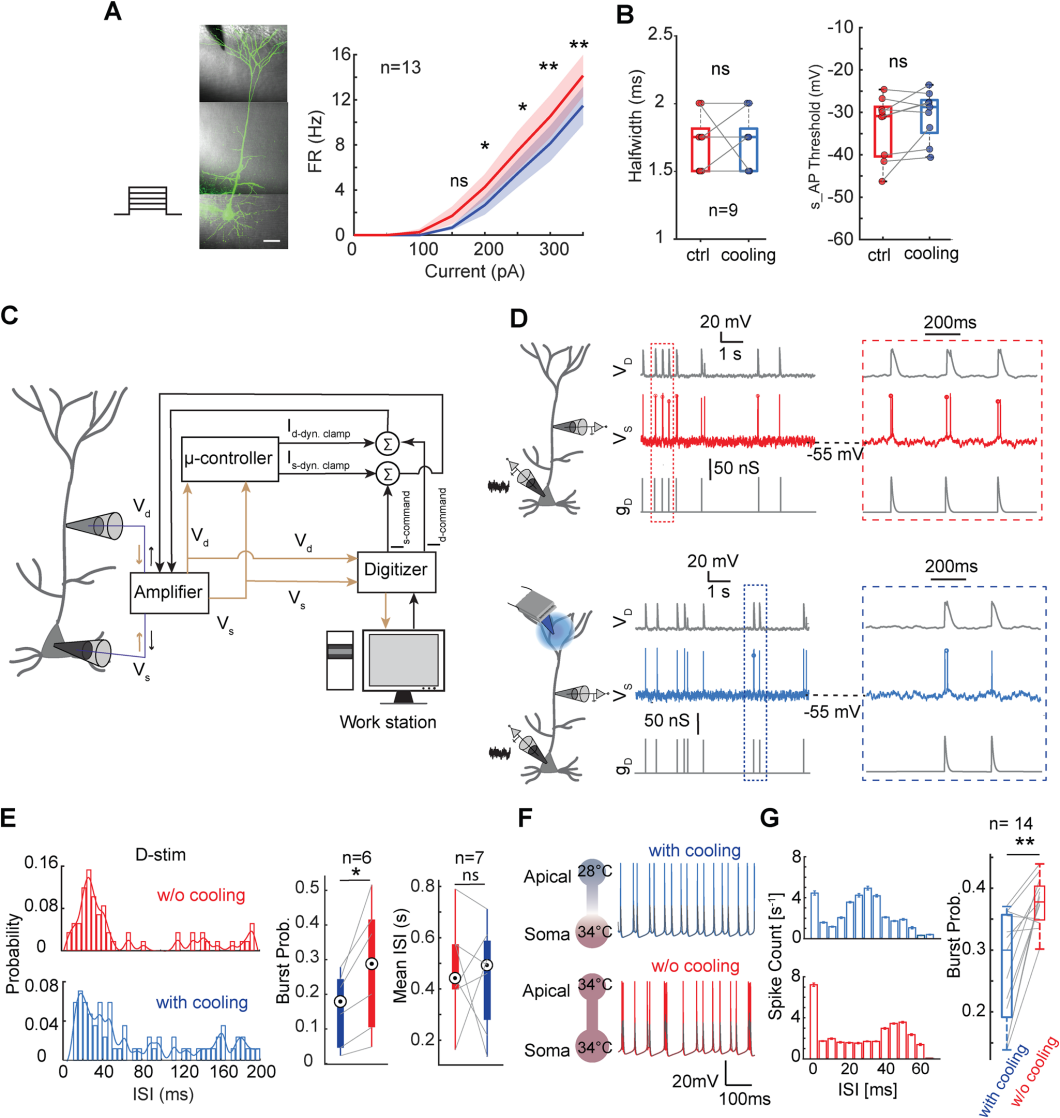
Temperature modulation across the tuft dendrite impacts L5 gain. (A) Cooling the tuft dendrite decreases the overall gain (^*^ *p*<0.05, ^**^ *p*<0.01, ^***^ *p*<0.001; Wilcoxon signed‐rank test). Scale bar: 50 µm. (B) The half‐width and spike threshold at the soma are not significantly affected by dendritic cooling (ns, *p* > 0.05). (C) Schematic of the dynamic clamp circuit that generates in vivo‐like conductance state through a feedback loop. (D) Cooling effect on the intrinsic burstiness when a subset of bAPs is paired with apical excitatory conductance without (top block) and with cooling (bottom block). (E) Histogram of burst probability as a function of the I.S.I in the presence of dendritic input with and without mild focal cooling. The horizontal axis represents dimensionless ISI and the threshold for burst is considered to be 50 ms, which corresponds to 0.1 on the dimensionless horizontal axis. (F) (left) Burst probability as a function of the temperature (Wilcoxon signed rank test, n = 6 neurons, *p* = 0.0313). (Right) The mean ISI (Wilcoxon signed rank test, n = 7 neurons, *p* = 0.6875). Note: a burst is defined as two consecutive spikes with the ISI smaller than 50 ms. (G) Spiking response of a three‐compartment biophysical model under dendritic focal cooling (top) and without cooling (bottom), gray traces indicate corresponding dendritic voltage. (H) Burst probability is reduced under apical focal cooling compared to physiological temperature (n = 14, the variance is due to imposed modulation in the dendritic geometry across models, see Methods for details, Wilcoxon signed rank test, *p* < 0.005).

Next, we examined how mild focal cooling of the tuft dendrite impacts gain. The F‐I curve (see Methods), with and without focal cooling across the tuft, revealed a reduction in neuronal gain (Figure 5A). Surprisingly, however, the spike half‐width (Figure 5B, left) and threshold (Figure 5B, right) did not change appreciably (n = 9 neurons, p > 0.05, Wilcoxon signed‐rank test), suggesting that spike structure remained unaffected and was not broadened by cooling the tuft dendrite as predicted by traditional models. This is while direct cooling of the soma increases the spike half‐width (Figure S19 n = 7 neurons, p = 0.0382, paired *t*‐test) while not changing the capacitance of the membrane (Figure S20, n = 7 neurons).

We thus surmised that changes in neuronal coding might underlie the shift in gain observed. We hence probed burst integration and inter‐spike intervals (critical for neuronal dispersion), including those under an in vivo‐like conductance state. We used a custom‐built dynamic clamp (Figure 5C,D) (see Methods) to maintain a high conductance state and incorporated BAC firing into the dynamic clamp to ensure the output reflected bursts. Bursts were identified when the inter‐spike intervals (ISI) were shorter than 50 ms (Figure 5E). Here, we observed that while the average firing rate was not perturbed, the burst probability reduced. Under cooling, the burst probability (number of bursts divided by total spikes) averaged 0.17±0.04, significantly lower than the 0.26±0.07 observed without tuft cooling (Figure 5F, left; p = 0.031, Wilcoxon signed‐rank test). However, the mean ISI did not differ significantly between cooling and non‐cooling conditions (Figure 5F, right, p = 0.687). These results indicate that mild focal cooling of the tuft reduces burstiness but does not significantly affect the average ISI.

To mechanistically corroborate this effect, we resorted to computational modeling (Figure 5G,H) using a multicompartment model of a layer 5 thick‐tufted neuron [83] (see Methods, and Figure S21A). In the model, we incorporated long‐term inactivation kinetics of apical dendritic Na^+^ channels and their temperature dependencies [76], and recapitulated the impact of temperature on frequency‐dependent attenuation of bAP amplitude (Figure S21A–C). To validate the temperature sensitivity in this model, we cooled the somatic compartment and observed a reduction in spike rate, consistent with previous reports [27, 28, 56] (Figure S21D). The three‐compartment biophysical model showed that focal cooling of the apical tuft dendrites alters the output spiking pattern in comparison to physiological baseline conditions without focal cooling at the tuft (Figure 5E). The mean firing rate was unchanged (with cooling: 34.23 ± 0.86 Hz, under physiological baseline temperature: 33.10 ± 1.03 Hz, mean ± std), but the burst probability (Figure 5G) reduced under a mere 5°C–6°C difference between tuft and soma (n = 14 (the variance is due to imposed modulations in the dendritic geometry across models, see Methods), Wilcoxon signed rank test, *p*<0.005). This effect was significantly impacted by the gating kinetics of Na^+^ channels and the temperature dependence of recovery from inactivation (Figure S21B–D). When the temperature dependence of Na^+^ channels was removed, the reduction in burst probability was abolished and increased above the baseline (Figure S21E). Together, this establishes a model in which K^+^ channels in the tuft are responsible for the increased excitability. However, the Na^+^ channel's dependence on temperature, as reflected in its recovery from inactivation, controls the burst probability and output rate. This suggests that a temperature‐induced degeneracy [76, 77] between K^+^ and Na^+^ channel dynamics controls L5 pyramidal neuron spiking, resulting in a reduction of burst‐dependent output gain and altered temporal structure of cortical output.

To test whether these dendritic mechanisms and model predictions manifest at the population level in vivo, we analyzed silicon probe recordings from layer 5 pyramidal neurons during focal cortical cooling. Consistent with the modeling results, cooling altered the temporal structure of neuronal firing without significantly changing the mean firing rate (Figure S22). Current source density analysis further revealed enhanced superficial current sinks during cooling, indicative of increased dendritic engagement. Together, the in vivo observations support the conclusion that focal cooling modulates burst‐dependent output and dendritic integration rather than globally suppressing neuronal excitability, providing experimental validation of the mechanisms proposed in Figure 5.

## 3 Discussion

Understanding how focal cooling influences neuronal output to impact cortical function is essential for unraveling the neural computations that underpin behavior. Layer 5 (L5) pyramidal neurons are uniquely positioned within the cortical circuit due to their distinct morphology, extensive connectivity, and key role in driving cortical output and sensorimotor integration, distinguishing them from the more superficial L2/3 neurons. Our results, combining in vivo (Figure 1) and ex vivo physiology (Figures 2, 3, 4, 5), strongly supports the notion that even modest cooling from the brain's surface, sufficient to induce behavioral change, can significantly impact L5 dendrites via changes in dendritic excitability (Figures 2 and 3), synaptic plasticity (Figure 4), and neural gain (Figure 5). This provides a cellular mechanism by which temperature modulates cortical output, highlighting why investigating L5 is critical for understanding the layered complexity of temperature‐based modulation.

From an in vivo perspective (Figure 1), we observed that focal cooling of the primary somatosensory cortex profoundly modulates sensorimotor integration with layer‐ and compartment‐specific effects. L5 pyramidal neurons in S1 are known to play a crucial role in whisker movement and tactile processing, making them a prime target to study how temperature affects behavior. Behaviorally, and although mice maintain task engagement (Figure 1F,G), cooling caused an increase in whisker phase concentration alongside reduced amplitude and delayed lick responses, indicating that the precision of active touch is disrupted (Figure 1I–L). At the same time, the basic whisking rhythm remained intact. These in vivo behavioral changes align well with our ex vivo observations that tuft and apical dendritic—not somatic—compartments of L5 neurons are selectively sensitive to cooling, linking cellular‐level modulation to tangible functional outcomes. Because dendritic integration in L5 neurons is essential for accurate processing of sensory inputs and for the transformation of whisker‐related signals into perceptual decisions [9], disruption at this dendritic level offers a clear mechanistic explanation for the impaired precision of touch observed here. Moreover, the dissociation between preserved whisker rhythm and impaired lick latency highlights the pivotal role of S1 circuits in refining sensory perception through precise temporal coordination, rather than merely driving sensory perception. Importantly, our measurements reveal a steep temperature gradient across cortical layers (Figure 1E) with only moderate cooling reaching the depths of L5 axon initial segments and proximal basal dendrites, pinpointing dendritic mechanisms within superficial L5 layers as likely drivers of the observed behavioral and electrophysiological effects, and suggest that the prevailing model of spike broadening and conduction velocity change is not the sole contributor to cooling‐induced behavioral change.

At the cellular level, cooling selectively enhanced dendritic excitability in the apical tuft of L5 pyramidal neurons while leaving basal dendritic excitability largely unaltered. This compartment‐specific effect was reversible and dependent on Kv4.2 potassium channel activity (Figure 2; Figure S7) pointing to a key molecular mechanism modulated by temperature. Increased input resistance and voltage attenuation along the apical dendrite during cooling (Figures 2 and 3) indicate heightened local excitability with reduced voltage spread to the soma, which likely contributes to a more compartmentalized dendritic integration (Figure 4). Importantly, we found that back‐propagating action potentials (bAPs), which influence burst firing and synaptic plasticity, show increased amplitude and integral area under cooling but suffer frequency‐dependent attenuation due to slowed recovery of sodium channels from inactivation (Figure 3). Thus, cooling paradoxically enhances initial bAP efficacy yet restricts high‐frequency burst propagation, providing a nuanced influence on neuronal output. These findings underscore how subtle temperature shifts can dynamically reconfigure dendritic computations and neuronal signaling.

These cellular dynamics translated into fundamental shifts in synaptic plasticity rules within dendritic compartments. Mild cooling induced robust spike‐timing‐dependent potentiation selectively in tuft dendrites, reversing the expected depression (Figure 4). Moreover, mild cooling only led to depression across basal dendrites under the same conditions. This differential modulation reflects the enhanced capacity of distal dendrites to integrate synaptic inputs and generate calcium spikes facilitated by improved bAP invasion during cooling. Additionally, low‐frequency plasticity induction protocols revealed that cooling potentiates synaptic efficacy at tuft dendrites but depresses basal dendritic synapses independent of temperature, highlighting compartmentalized roles for temperature in synaptic strengthening. The Kv4.2 channel emerged again as a critical modulator, linking dendritic excitability and plasticity under thermal modulation. These results point toward temperature‐dependent mechanisms as potential modulators of learning and memory processes, with implications for state‐dependent cognitive flexibility and adaptation.

Functional consequences of these dendritic changes were reflected in alterations of neuronal gain and firing patterns (Figure 5). Cooling reduced the gain of L5 pyramidal neurons without affecting their spike shape or threshold, a phenomenon not explained by classical models that predict spike broadening during cooling (Figure S19). Instead, dynamic clamp experiments and biophysical modeling revealed that cooling selectively decreases burst probability while preserving overall firing rate, which is attributable to the interplay between increased dendritic excitability mediated by potassium channels and the temperature sensitivity of sodium channel kinetics. This reduced burstiness could impact cortical output by modulating the temporal coding of sensory inputs and may underlie the observed behavioral deficits in whisker coordination. This mechanistic insight into burst modulation reveals how local temperature shifts can fine‐tune neural coding strategies and affect cortical information processing efficiency.

Overall, these results depict a sophisticated and compartmentalized mechanism by which mild focal cooling tunes cortical network function. Cooling favors dendritic excitability and plasticity in apical tuft segments via Kv4.2 channels, while simultaneously constraining burst firing through temperature‐dependent sodium channel inactivation, together reshaping neuronal gain and output patterns in L5 pyramidal cells. Behaviorally, these cellular effects manifest as a disruption of active touch precision without abolishing basic motor functions, implying that cortical temperature gradients may dynamically regulate sensorimotor integration. This work advances our understanding of how local cortical temperature modulates the biophysical properties of dendrites and reconfigures network dynamics, serving as a potential physiological or pathophysiological modulatory mechanism in somatosensory processing and plasticity. Given the ubiquitous nature of cortical temperature fluctuations during various brain states, sleep, and pathological conditions, these findings have broad implications for understanding the influence of thermal dynamics on cortical computations and behavior.

## 4 Conclusion

This study provides evidence that mild, spatially precise focal cortical cooling modulates cortical computation by selectively engaging dendritic mechanisms in layer 5 pyramidal neurons. By inducing a steep but localized temperature gradient (∼4°C), suggesting limited influence on axonal conduction and infragranular somatic compartment, focal cooling enhances apical tuft impedance and modulates input‐output transformations while impairing dendritic Na^+^ channel recovery, paradoxically weakening somato‐dendritic coupling. This compartment‐specific modulation alters neuronal gain and overall cortical output. In behaving animals, these effects manifest as degraded tactile precision accompanied by preserved rhythmic motor activity, linking microscale dendritic modulation to mesoscale sensory‐motor computation. Collectively, these findings establish high‐resolution focal cooling as a reversible and physically grounded neuromodulatory approach that leverages intrinsic dendritic thermal sensitivity to dissect and dynamically tune cortical processing. This study provides a mechanistic framework for the effect of focal cooling on the brain.

## 5 Materials and Methods

### 5.1 Acute Slice Preparation

All experimental procedures were conducted in accordance with the guidelines set forth by the NIH and Purdue Institutional Animal Care and Use Committee (IACUC) (protocol number 1910001968). All physiological solutions were fully oxygenated (95% O_2_ and 5% CO_2_), pH adjusted to 7.3–7.4, and osmolarity maintained between 300 and 310 mOsm unless stated. Adult C57BL/6 (Jackson Laboratory) mice (both male and female, 8–12 weeks of age) were deeply anesthetized with 3%–4% isoflurane followed by trans‐cardiac perfusion with ice‐cold NMDG cutting solution [84] consisting of (in mM): 92 NMDG, 30 NaHCO_3_, 1.2 NaH_2_PO_4_, 20 HEPES, 2.5 KCl, 25 glucose, 5 sodium ascorbate, 3 sodium pyruvate, 2 thiourea, 0.5 CaCl_2_, 10 MgCl_2_, 5 N‐acetyl‐L‐cysteine, before decapitation. Coronal slices (300–350 µm thickness) were prepared using a vibratome (Leica VT1200S) in 0°–4°Celsius NMDG cutting solution. Brain slices were then allowed to recover in 34°C NMDG cutting solution with gradual perfusion of 0.25 to 0.5 mL Na^+^ rich NMDG solution (2 m NaCl in NMDG cutting solution) over 6–10 min depending on the mouse age [85] and transferred to room temperature HEPES artificial cerebrospinal fluid (ACSF) holding solution consisting of (in mm): 92 NaCl, 2.5 KCl, 1.25 NaH_2_PO_4_, 30 NaHCO_3_, 20 HEPES, 25 glucose, 5 sodium ascorbate, 3 sodium pyruvate, 2 thiourea, 2 CaCl_2_, 2 MgCl_2_, 5 N‐acetylL‐cysteine for at least 1 h prior to recording.

### 5.2 Electrophysiological Recording

Slice recordings followed protocols previously established [26]. In short, slices were transferred to a chamber, continuously superfused with oxygenated ACSF, and visualized with an upright two‐photon microscope (Bruker Nano, Madison, WI) comprising of an Olympus BX51WI body (Olympus, Tokyo, Japan) fitted with infra‐red (IR) Dodt‐gradient‐contrast (DGC) optics, an IR sensitive camera (IR‐2000, Dage‐MTI, Michigan City, IN), and a 40x water immersion objective (0.8 NA, Nikon USA). Recordings were performed in 35°C recording ACSF consisting of (in mm): 125 NaCl, 3 KCl, 25 NaHCO_3_, 1.25 NaH_2_PO_4_, 25 glucose, 3 sodium pyruvate, 1 sodium ascorbate, 1.3 CaCl_2_, 1 MgCl_2_. 4 to 8 mΩ borosilicate patch pipette (Sutter Instruments, CA, USA) was pulled using a P1000 pipette puller (Sutter Instruments, Novato, CA), filled with internal solution containing (in mm): 130 potassium gluconate, 7 KCl, 10 HEPES, 5 NaCl, 35 sucrose, 2 MgSO_4_, 2 sodium pyruvate, 4 Mg‐ATP, 0.4 Tris GTP, 7 phosphocreatine disodium (pH 7.3, osmolarity 290mOsm). 25 µm Alexa 594 or 100 µm Alexa 488 was used for two‐photon structural imaging, 200 µm fluo‐4 was used for two‐photon Ca^2+^ imaging. For targeting layer 5 pyramidal neurons, we have targeted large, thick‐tufted somata located 600–900 µm below the pial surface, consistent with layer 5b. Cells were further identified by their morphological features under infrared differential interference contrast (IR‐DIC) optics. To confirm cell identity, we assessed intrinsic firing properties in response to somatic current injection; neurons included in this study exhibited either intrinsically bursting or regular spiking patterns. Fast‐spiking interneurons and other non‐pyramidal cell types were excluded from analysis. L5 pyramidal neurons that were patch‐clamped had typical resting membrane potentials between −60 and −70 mV without current injection. In cases where the spontaneous membrane potential was more negative (e.g., ∼−75 mV), we applied a small steady depolarizing holding current (typically 50–100 pA, adjusted per cell) to maintain the membrane potential within a consistent target range (−60 to −70 mV) during recordings. Whole‐cell recordings were made using a Multiclamp 700B, (Molecular devices, San Jose, CA), Bessel‐filtered at 4 kHz, and digitized at 4 to 20 kHz using a Digidata 1550B interface (Molecular devices, San Jose, CA) and winwcp software. Slice recordings were performed in a temperature‐controlled recording chamber with the bath temperature set to 35°C using an inline heating system. Bath temperature was verified using an external thermometer at the start of each experiment and re‐checked following focal cooling protocols. Adjustment of the cooling probe setpoint did not affect the bulk bath temperature. No appreciable bath temperature drift was observed during the course of recordings.

### 5.3 Focal Cooling

The cooling device is consisted of a 250 µm diameter silver wire (A‐M systems, #781000; thermal conductivity 406 W/mK) wrapped with 25 µm graphene sheet (graphene‐supermarket: Conductive Graphene Sheets, thickness: 25 mm; thermal conductivity: 1300–1500 W/m in x‐y plane and 13–15 W/m in z plane) and insulated with a polyimide tube (1.1 mm diameter, Accu‐Glass Product Inc., #111217) [86]. The polyimide tube was further sealed with adhesive epoxy (Newark, TBS20S; thermal conductivity: 1.1 W/m.K) in both ends to create a contained air insulation around the silver wire and avoid ACSF leakage into the tube. Only 2 mm of the silver wire was protruding out of the insulation layers to be exposed for cooling the tissue. The silver wire was coiled on one end and glued with Arctic Silver Adhesive (Custom Thermoelectric, TG‐AS5‐12G) to the cold side of a Peltier device (Custom Thermoelectric, 02301–9B30‐32RU6A) which was itself getting cooled on its hot side by a homemade aluminum active heat sink. To have a better spatial resolution in cooling the spots on the tissue, the tip of the silver probe was sharpened by electro etching with sodium nitride solution to deliver a tip size of 10–30 µm. Prior to starting electrophysiological experiments, a k‐type temperature sensor (Omega, 80 µm wires, product number 5SC‐TT‐K‐40‐72) was used to calibrate the temperature of the probe tip and the tissue once the probe was into the tissue. The solution temperature in the chamber was stable at around 35°C, and the tip could be cooled down to 29.6°C in the best case. Prior to the experiment, the thermocouple was calibrated against the tissue temperature. Repeated measurements showed stable readings with trial‐to‐trial variability typically <0.3°C–0.5°C. Based on manufacturer specifications and in situ calibration, instantaneous measurement uncertainty during rapid temperature transitions is on the order of ∼0–2°C; however, once the temperature reached steady state, the effective measurement error was substantially lower (typically ≤0.5°C under our recording conditions). Because only the welded junction generates the thermoelectric signal and the leads are PFA‐insulated, the recorded values reflect local tissue temperature at the junction rather than the cooling probe itself. These measurements therefore provide reliable estimates of local temperature gradients within the 5°C–6°C cooling range used in vitro.

### 5.4 Surgical Procedure

All experimental procedures were conducted in accordance with the guidelines set forth by the NIH and Purdue Institutional Animal Care and Use Committee (IACUC) (protocol number 1910001968). Mice were first deeply anesthetized with 3%–4% isoflurane. During surgery, anesthesia was maintained at 1%–1.5% isoflurane with an oxygen flow rate ∼0.1 L/min. An infrared warming pad (Kent Scientific) was used to maintain the body temperature. Carprofen and Dexamethasone (0.6 mg/kg body weight) were injected subcutaneously, and lidocaine was injected under the scalp after the induction of anesthesia. Eye ointment was applied, and the scalp was shaved and sanitized before the incision. After scalp removal, we briefly applied 3% hydroperoxide to remove excess tissue and immediately cleaned the skull with saline perfusion. Vetbond (3 m) was used to seal the tissue surrounding the skull region. We then used dental cement to adhere a custom‐designed titanium headplate (Parkell) to the skull centered over the left barrel cortex (1.5 mm posterior to bregma and 3.5 mm lateral to midline). Once the cement solidified, we used a dental drill to make a small craniotomy (∼1–2 mm diameter) over the primary somatosensory cortex corresponding to the C1 barrel column, guided by intrinsic signal imaging and stereotaxic coordinates. Animals were given 3–7 days to habituate to the headplate. 1 day prior to recordings, we trimmed all but the C1 whisker and performed intrinsic optical imaging to locate the C1 barrel (described below). Upon completion of the surgery, we covered the cranial window with a small piece of surgical foam (Surgifoam) soaked in ACSF and transferred the animal to the recording rig, where it remained head‐fixed for the duration of the experiment.

### 5.5 Mouse Behavior

Two days after head plate implantation, mice were given the opportunity to run on a circular treadmill for 1 h daily until they fully habituated to the apparatus and were able to maintain a steady running pace. This facilitated volitional running and active whisking during behavior. Mice were water‐deprived and trained in a goal‐directed whisker detection task, in which they were required to lick in response to tactile surface contacts in order to obtain a water reward. To minimize extraneous sensory input, an opaque flag was placed over the eye, and white noise was broadcasted throughout the task. The circular treadmill was attached to a rotary encoder to measure running distance. Whiskers were imaged during the trial window with a high‐speed camera at 250 Hz. The mice were trained/recorded for one 60 min session per day. During the session, a trial was initiated when the animal started running. Each trial contains a 1.8 s baseline period (before the pneumatic piston moves to the whisking field), a 2 s sampling period (when the pneumatic piston is within with whisking field), and a 2 s post‐sampling period. During the sampling period, a pneumatic piston moved the tactile surface into the active whisking field, where the mouse actively located the object. At the end of the sampling period, the surface was retracted outside the whisking field. If the animal licked the water spout during the sampling period, it indicates a correct detection (hit), and the animal receives a water reward 200 ms after the piston retracts. The inter‐trial interval was not fixed as the trials were initiated by the animal running. If the animal sustained the running after a trial finishes, a minimum inter‐trial interval of 1 s was enforced to reduce the chance of impulsive licking. The cooling/recording sessions started once the animal learned the task (hit rate higher than 80%). During a recording session, the cooling was started at around 25 min and stopped at around 45 min. The trials during both the baseline period (the 20 min before cooling) and the recovery period (the 20 min after cooling) were pooled in the analysis to remove any confound caused by loss of engagement by the animal.

### 5.6 Whisker Imaging and Tracking

Whiskers were imaged using a high‐speed (250 fps) infrared camera (photonfocus DR1) through a mirror angled at 45° under IR illumination. Imaged videos were synchronized with mouse licking onset via an external trigger using an Arduino microcontroller and recorded on an Intan 512 controller. DeepLabCut was used to track whisker movement and curvature [64]. About 100 frames from each recording session were labeled manually, spanning diverse whisker positions. The neural network was trained for at least 100‐k iterations, and the final labels were manually verified for accuracy.

### 5.7 Extraction of Active Whisking Kinematics

Whisker position was determined by measuring the angle between the vertical axis of the video frame and a line connecting a reference point on the mouse's nose to a labeled point on the whisker. The same labeled point was used across both intact and trimmed whisker conditions. To extract the whisker envelope, peaks and troughs of the position trace were interpolated using Akamai spline interpolation. The whisker offset was defined as the mean of the upper and lower envelope, and the amplitude was calculated as half the envelope's width. Whisker phase was derived by first bandpass filtering the position signal between 5 and 50 Hz (MATLAB), then applying the Hilbert transform and extracting the instantaneous phase. In this phase convention, 0° and −π represent the most retracted whisker position, while π corresponds to the most protracted position. Whisker phase concentration was quantified as the magnitude of the mean resultant vector of the instantaneous whisker phase distribution across time within a trial, with values near 1 indicating tightly clustered (phase‐locked) whisking and values near 0 indicating dispersed or irregular phase.

### 5.8 In Vivo Silicon Probe Recordings

Male and female C57BL/6J mice (12–24 weeks old) were used for the in vivo silicon probe recordings with 64‐channel silicon probes (Masmanidis Lab in UCLA, 64F and 64 m). The animals were habituated and trained on the tactile detection task prior to the recording sessions. After anesthetizing the animal, a small cranial window (around 2 mm in diameter) was made with a dental drill on the skull above the target barrel, identified via intrinsic optical imaging. The silicon probe was inserted at an angle of 10 degrees and a speed of 2 µm/second, and the cooling probe was positioned next to the silicon probe. Signals were digitized at a bandwidth of 0.1–10 kHz and sampled at 20 kHz (Intan RHD, USA). In the optogenetic experiments, the mice were injected with AAV9‐CaMKIIa‐aChR2‐mCherry (addgene, 26975) in S1 three weeks before training, and a blue fiber‐coupled LED (470 nm, Thorlabs, M470F4) was positioned above the targeted barrel (around 1 mm from the brain surface). After the recording session, we subjected electrical signals to processing, including sorting action potentials from individual neurons. This sorting was accomplished using Kilosort4 (https://github.com/MouseLand/Kilosort). Neurons were evaluated based on waveform characteristics, stability of firing rates observed throughout the recording session, and auto‐correlograms with custom Matlab codes.

### 5.9 Go/No‐Go Tactile Discrimination Task

To assess whisker‐based tactile discrimination, a head‐fixed Go/No‐Go task was performed using two adjacent whiskers (C1 and D1). Mechanical stimuli were delivered with independently controlled pneumatic pistons positioned within the active whisking field and directed to either the C1 or D1 whisker. C1 touch served as the Go stimulus, for which animals were trained to lick the water spout to obtain a water reward, whereas D1 touch served as the No‐Go stimulus, for which animals were required to withhold licking. Animals were first trained by classical conditioning with the Go stimulus, then exposed to the No‐Go stimulus, and finally advanced to operant training in which they learned to withhold licking during No‐Go trials. Behavioral performance was quantified using the sensitivity index 𝑑′, calculated from hit rate and false alarm rate. Whisker kinematics were recorded simultaneously with high‐speed infrared imaging and analyzed using DeepLabCut [64] to quantify whisker position and curvature during task performance. For cooling experiments, the focal cooling probe was positioned above the cortical region corresponding to the C1/D1 barrel junction, and behavioral performance was compared across control, cooling, and recovery conditions.

### 5.10 Thermal Transport Simulation

Numerical simulations were performed to model a 2D thermal transport for the probe passing through ASCF and inserted in the slice using a finite element‐based COMSOL Multiphysics 5.6. The effect of the diameter and length of the silver wire was investigated to find the optimal design for cooling the slice, and the results are summarized in Figure S5.

### 5.11 Field Evoked Stimulation

Focal electrical field evoked stimulation was performed via a theta‐glass (borosilicate; Hilgenberg) pipette pulled using a P1000 pipette puller (Sutter Instruments, Novato, CA), and filled with the recording ACSF solution. The pipette tip was located at approximately 5–10 µm from the target dendritic segment guided by the fluorescent image of the dendrite. A biphasic current pulse with 0.1 ms duration was injected through the electrode at various intensities (1–5 µA) via stimulus isolator (ISO‐Flex; AMPI). The effectiveness and localization of the stimulation were verified by measuring the Ca^2+^ intensity resulting from the stimulus pulses. The baseline recordings were performed at a frequency of 1 Hz, and the low‐frequency plasticity induction was performed at 0.1 Hz. For the STDP induction, single EPSPs were paired with single spikes with a delay of +10 ms. Each pairing was repeated 50 times.

### 5.12 Pharmacology

For the plasticity experiments, gabazine (500 µm; Sigma) and metabotropic gamma‐aminobutyric acid receptor blockers (10 mm CGP‐55845) were added to the recording ACSF solution. In specific experiments, NMDAR blocker (100 mm APV; Tocris Bioscience) and Kv4.2 subunit channel blocker (0.5 mm heteropodatoxin‐2; Alomone) were added to the ACSF perfusion solution 20 min before the start of the recording session. After the experiments, the recording ACSF solution containing the pharmacological blockers was washed in through a perfusion system, and the next recording session was performed 10–20 min after the pharmacological blocker wash‐in.

### 5.13 Dynamic Clamp

A microcontroller‐based circuit board was used for performing dynamic clamp experiments as previously described^40^. The module was controlled with custom‐written codes (Arduino, Processing). First, the linear input‐output relationship of the amplification circuit was measured with a DC voltage source (E3631A Agilent Technologies, Santa Clara, CA) and oscilloscope (Keysight, Santa Rosa, CA), and then verified with a model cell (Molecular devices, San Jose, CA). The working principle is as follows: the microcontroller reads the output of the amplifier, and then A/D converts the amplified membrane voltage, computes the current based on differential equation models, and D/A converts the current, which is then summed with the current command from the Digidata 1550B interface (Molecular devices, San Jose, CA). The ODEs are solved using the forward Euler method.

The Ornstein–Uhlenbeck process‐based point conductance model [87] mimics background synaptic inputs under a noisy in vivo‐like state. We used this point conductance model instead of a Poisson train of synaptic inputs [88] to ensure higher variability in the amplitude of EPSPs and IPSPs, better reflecting the background synaptic inputs with different synaptic strengths. The dynamic conductance is computed with the following differential equations (Equations (1), (2), and 3) (χ_1_ and χ_2_ are two independent random variables following a unit normal distribution).

(1) $$I_{syn}\;\left(t\right)=g_{e}\;\left(t\right)\cdot \left(V_{m}\left(t\right)-E_{e}\right)+g_{i}\left(t\right)\cdot \left(V_{m}\left(t\right)-E_{i}\right)$$

(2) $$\frac{dg_{e}\left(t\right)}{dt}=\frac{{\bar{g}}_{e}-g_{e}\left(t\right)}{\tau_{e}}+\sqrt{D_{e}}\cdot \chi_{1}\left(t\right),\chi_{1}∼N\left(0,1\right)$$

(3) $$\frac{dg_{i}\left(t\right)}{dt}=\frac{{\bar{g}}_{i}-g_{i}\left(t\right)}{\tau_{i}}+\sqrt{D_{i}}\cdot \chi_{2}\left(t\right),\chi_{2}∼N\left(0,1\right)$$

In our experiment, τ_𝑒_ = 2.8 ms, τ_𝑖_ = 8.5 ms, E_e_ = 0 mV, E_i_ = −80 mV, were chosen based on the physiological properties of α‐amino‐3‐hydroxy‐5‐methyl‐4‐isoxazolepropionic acid (AMPA) and γ‐Aminobutyric acid (GABA) channels. The mean of noisy excitatory conductance (g_e_) varies between 2 and 4 nS for different amounts of spontaneous background firing rate, and g_i_ equaled 2–9 nS. Conductance values agreed with previous literature, and this range helped match the background firing rates to those observed in vivo (2–20 Hz), but avoided a hyperexcitable state. The noise diffusion coefficients of the excitatory (D_e_) and inhibitory conductance (D_i_) were scaled to match voltage fluctuations normally observed in vivo (∼5 mV RMS). These terms, which contribute to the “noisy” nature of dynamic conductance, were in line with previous literature [87] and this ensured the fast‐fluctuations in the background were preserved.

To mimic coincident apical synaptic inputs and bAPs (Figure 5C–E), a double exponential apical excitatory conductance (G_max_ = 50 to 70 nS, E_e_ = 0 mV, τ_1_ = 1 ms, τ_2_ = 10 ms) was applied through the dendritic dynamic clamp once the dendritic voltage exceeded the bAP threshold (‐40 mV to ‐15 mV dependent on the dendritic patch location) in a close‐loop manner.

(4) $$I_{apic}\;\left(t\right)=g_{apic}\;\left(t\right)\cdot \left(V_{apic}\left(t\right)-E_{e}\right)\;$$

(5) $$\frac{dg_{apic}\left(t\right)}{dt}=G_{max}\;*\left(dt\frac{-1}{\tau_{2}}-dt\frac{-1}{\tau_{1}}\right)\;$$

### 5.14 Two‐Photon Imaging and Glutamate Uncaging

A laser‐scanning microscope (Bruker Nano, Madison, WI) with dual galvanometric mirrors and two femtosecond pulsed lasers (Spectra Physics, Insight X3 and Mai‐Tai) was used to perform two‐photon imaging and glutamate uncaging. Laser beam intensities were independently controlled with electro‐optical modulators (model 350‐50; Conoptics, USA). The imaging beam (set to 810 nm for Alexa 594 and fluo‐4, and 920 nm for Alexa 488) was merged with the uncaging beam (720 nm) using a 760 nm long‐pass dichroic mirror. During Calcium (Ca^2+^ imaging, line scans were performed across the spine head and the adjacent branch with 8–10 µs dwell time (400–800 Hz line rate) and 3–5 mW laser power at the objective focal plane. Structural imaging was performed at 5–8 µs dwell time. For glutamate uncaging with 4‐Methoxy‐7‐nitroindolinyl‐caged‐L‐glutamate (MNNI‐glu, Tocris, MN, USA), 2.5 mm MNI‐glu was diluted in freshly prepared recording ACSF and applied to the bath through a circulating pump. At these concentrations, there was no epileptiform‐like activity. The uncaging dwell time was 0.5 ms, and the laser power needed for uncaging ranged from 20 to 30 mW. At these powers, no visible photodamage occurred. Baseline fluorescence of both channels was continuously measured to assay any damage. Ca^2+^ transients were also measured to ensure spines were still functionally active with no loss in physiological response. EPSP time course and changes in resting membrane potential following repeated stimulation were also assayed as indicators of any photodamage. Table‐top hard shutters were used to avoid exposure and any off‐target uncaging. Ca^2+^ signals were expressed as DF/F (calculated as (F—F_baseline_)/F_baseline_) [26]. Data was collected from dendrites at least 30 µm below the surface of the slice and was not prematurely cut off before termination.

### 5.15 Three‐Compartment Biophysical Model

The simplified biophysical model of L5 PNs consisted of three different compartments: soma‐AIS, proximal apical dendrite, and distal apical dendrite. The ion channels and corresponding parameters in each compartment are listed in Table S1 for further details. To mimic the diversity of morphology and intrinsic ion channel distribution across the L5 PN population, the ratio between the total membrane surface area of each compartment, as well as the distance between the dendritic compartments and the soma, was assigned with 5% jitter around the average value in Table S1. The distance between the proximal apical dendrite and the soma was kept larger than 210 µm to ensure intrinsic burstiness. Bursts were defined as two or more spikes occurring with 20 ms time interval or less. The differential equations were solved with the forward Euler method with an interval of 0.05 ms. The dendritic Na^+^ channel (nad) model was adapted from previous studies [76, 77], the Q10 value was set to 2.3 for all ion channels. The code implementing this simulation is available on github: https://github.com/shulanx1/simpl5pn_temperature_modulation.

### 5.16 Statistical Analysis

Data are reported as mean ± SEM unless otherwise stated. Statistical analyses were performed using MATLAB. Prior to applying parametric tests, assumptions of normality were evaluated. For paired comparisons (e.g., within‐neuron or within‐animal measurements), normality of paired differences was assessed. For unpaired comparisons, normality of group distributions was assessed. For analyses involving repeated measurements (repeated‐measures ANOVA and linear mixed‐effects models), normality of model residuals was evaluated. Parametric tests (t‐tests, ANOVA, repeated‐measures ANOVA, and linear mixed‐effects models with ANOVA‐style F‐tests) were used when assumptions were met. Linear mixed‐effects models were employed when appropriate to account for repeated measurements across neurons, animals, or sessions. All statistical tests were two‐sided with a significance threshold of α = 0.05.

For analyses involving multiple comparisons, global tests (ANOVA or linear mixed‐effects models) were first used to assess overall effects. Post‐hoc comparisons were restricted to a priori planned contrasts based on experimental hypotheses; therefore, no additional correction for multiple comparisons was applied unless otherwise stated. Box plots display the median (50th percentile) and interquartile range (25th–75th percentiles); whiskers denote the full data range unless otherwise stated.

### 5.17 Data, Materials, and Software Availability

Upon acceptance, all additional data will be uploaded to Zenodo, and the code will be uploaded to GitHub repositories, wherever applicable. The Zenodo link with the currently presented data in the manuscript is https://doi.org/10.5281/zenodo.14020289. This link will be made live upon acceptance with the entire data set Link to code with biophysical simulations https://doi.org/10.5281/zenodo.14020643. Dynamic clamp board https://doi.org/10.5281/zenodo.11303939

## Author Contributions

M.H.M., S.X., and K.J. designed the study and experiments. M.H.M. performed all experiments. S.X. assisted with experiments. M.H.M., S.X., and K.J. analyzed and interpreted data. M.H.M. and K.J. wrote the manuscript. All authors edited the manuscript. K.J. provided overall guidance and funding for the study.

## Conflicts of Interest

The authors declare no conflicts of interest.

## Supporting information




**Supporting File**: advs75106‐sup‐0001‐SuppMat.docx.

## Data Availability

The data that support the findings of this study are openly available in Zenodo at https://doi.org/10.5281/zenodo.14020289, reference number 14020289.
