# Supplementary material for: Mild Focal Cooling Decouples Dendrites to Reconfigure Cortical Output
Source: Adv Sci (Weinh). 2026 Apr 9;13(34):e20773. doi: 10.1002/advs.202520773 (PMC13285124; doi:10.1002/advs.202520773)
Supplement: Supplementary file 1 — Supporting File: advs75106‐sup‐0001‐SuppMat.docx. [file ADVS-13-e20773-s001.docx]

**Supporting Information for**

**Mild Focal Cooling Decouples Dendrites to Reconfigure Cortical Output**

Meisam Habibi Matin, Shulan Xiao, and Krishna Jayant

Corresponding author: [kjayant@purdue.edu](mailto:kjayant@purdue.edu)

**This PDF file includes:**

Figures S1 to S22

Tables S1

SI References


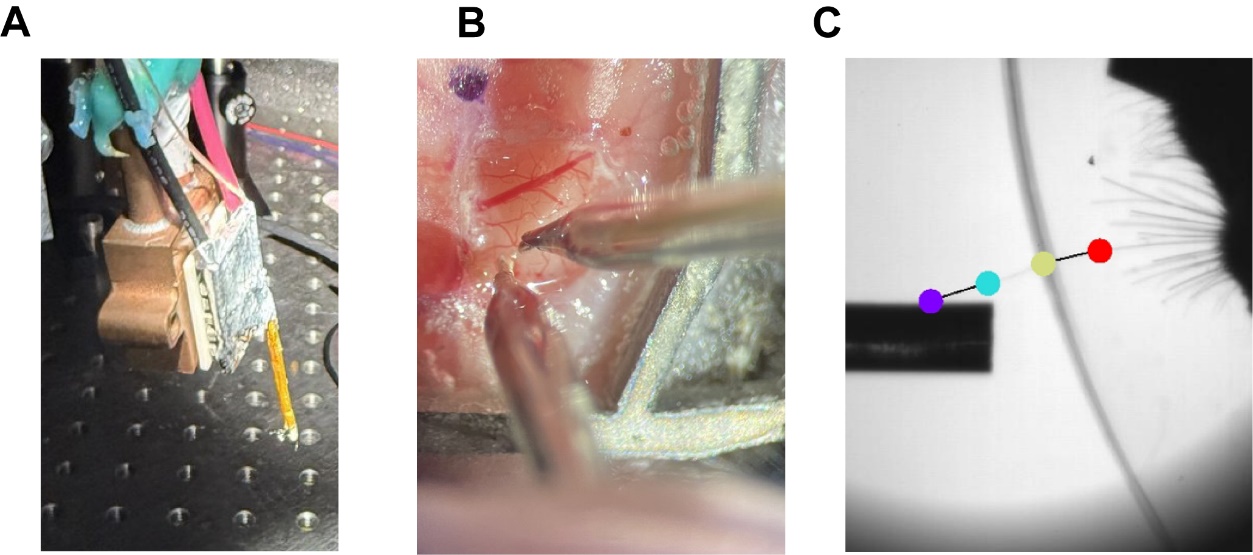


**Fig. S1.** **Cooling probe implementation in in vivo experiments**

**(A)** A photograph of the cooling device with the insulated probe connected to the thermoelectric Peltier and the heat sink. **(B)** Photograph of the implantation of the temperature sensor probe and the cooling probe to measure the temperature at different depth across the cortex. **(C)** Example frame from behavioral tracking showing active whisker contacts with the piston. Colored markers denote labeled whisker points identified using DeepLabCut for quantifying whisker kinematics during experiments.


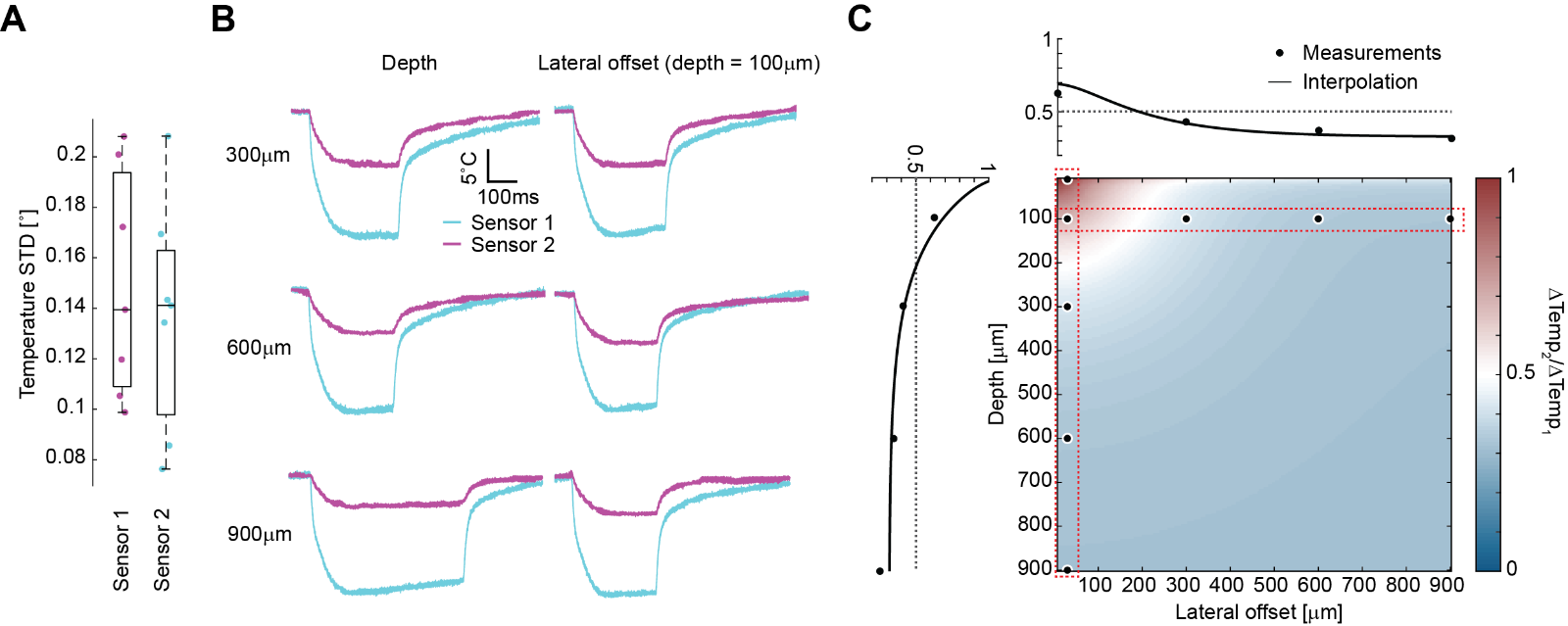


**Fig. S2. 2D map of the cooling effect with in vivo focal cooling on the brain surface**

**(A)** The standard deviation of temperature measurements on both sensors at physiological temperature (n = 7 repetitions). **(B)** Representative temperature measured at the cooling probe tip (sensor 1, cyan) and in the brain tissue with various axial and lateral distance to the probe tip (sensor 2, magenta).


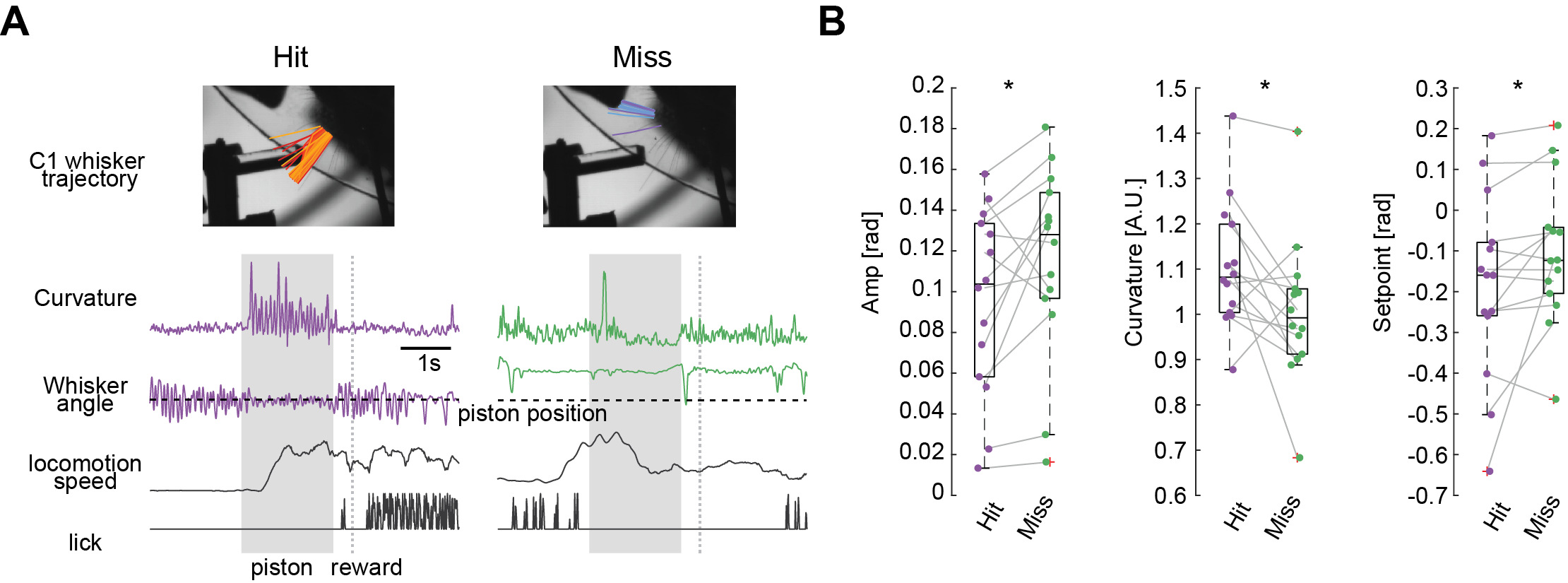


**Fig. S3. Whisking kinetics are correlated with tactile detection performance**

(A) C1 whisker trajectory, curvature, angle, locomotion and lick in an example hit trial and miss trial. (B) The whisking amplitude, curvature and whisker setpoint (middle point of the whisking range) during hit and miss trials (n = 14 sessions from 4 animals, Wilcoxon signed rank test, * p < 0.05).

**
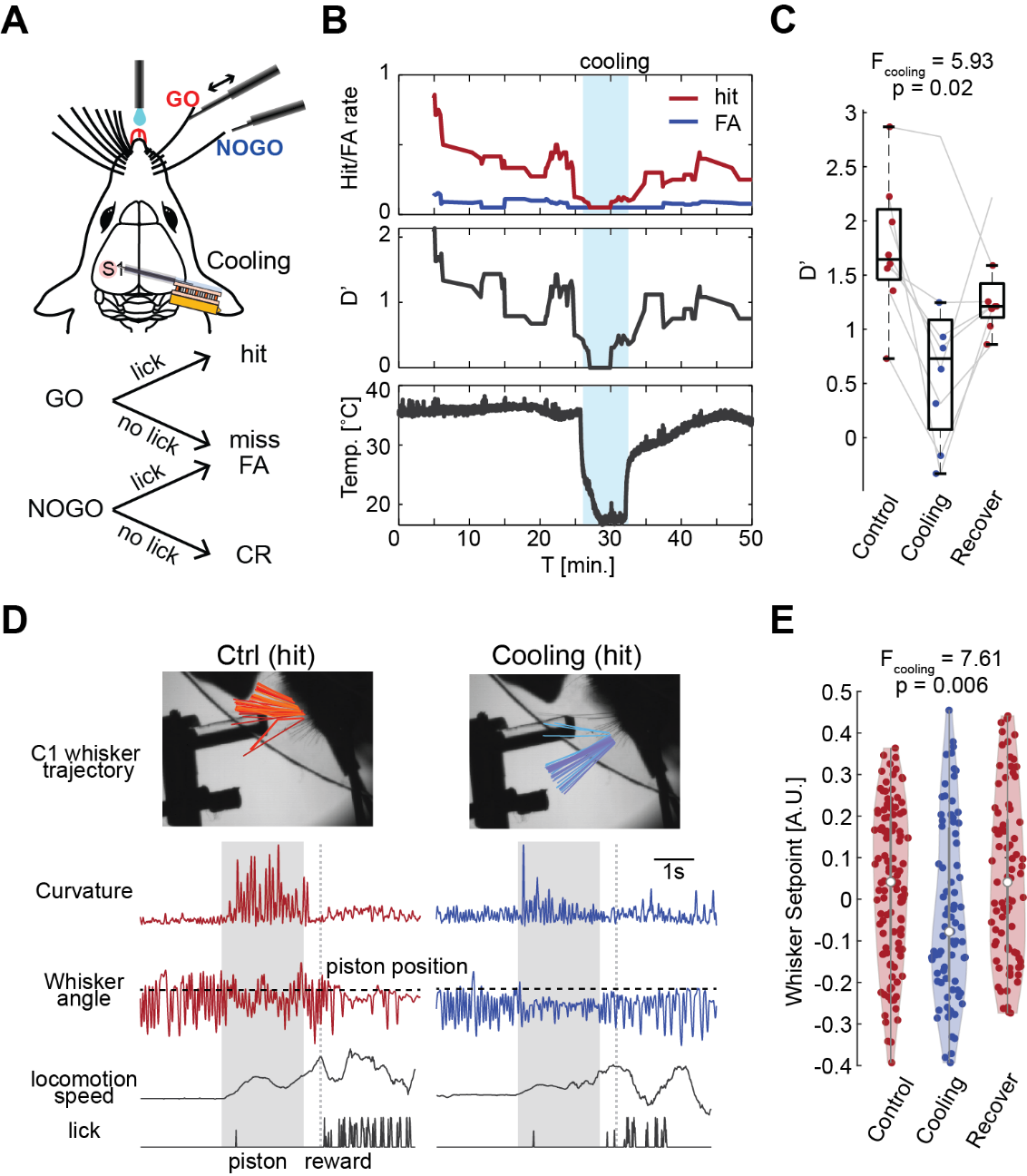
**

**Fig. S4. Cooling impairs the tactile discrimination performance**

**(A)** The two-whisker discrimination task. **(B)** The hit rate, false alarm (FA) rate and D’ in an example session. The blue box indicates cooling. **(C)** The D’ value under control, cooling and recovery from cooling (n = 8 sessions from 5 animals, one-way ANOVA with Tukey-Kramer post-hoc test). **(D)** C1 whisker trajectory, curvature, angle, locomotion and lick in example hit trials under control and cooling. **(E)** The whisker setpoint under control, cooling and recovery from cooling (n = 4 sessions from 2 animals, each individual data point reflects one trial. One-way ANOVA with Tukey-Kramer post-hoc test).


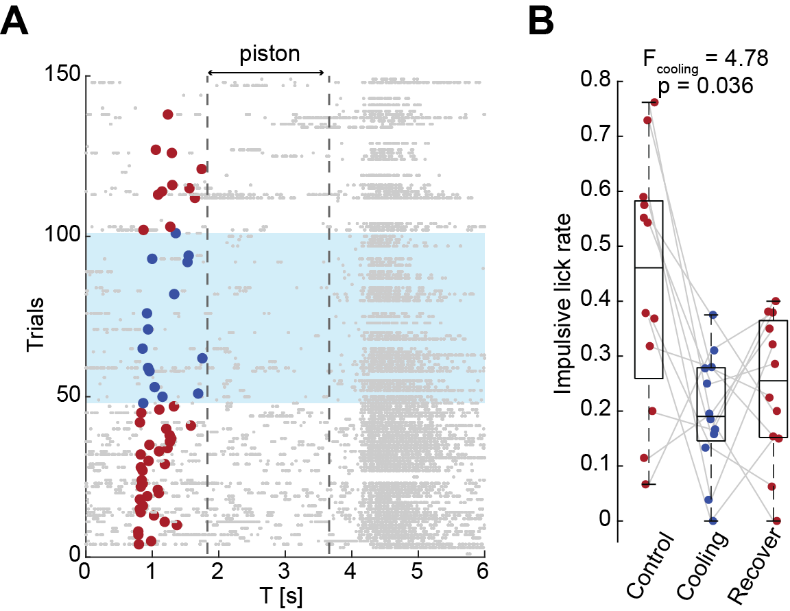


**Fig. S5. Cooling reduces the baseline spontaneous lick rate in the pre-stimulus window.**

**(A)** Example raster of licking events across trials during the whisker detection task. Each dot indicates an individual lick. Trials are aligned to stimulus timing. The dashed vertical lines indicate the time window during which the piston entered the whisking field. The red and blue dots indicate the first lick event during the 1 s window before the piston stimulus (spontaneous lick events). The blue shaded region indicates the cooling trials. **(B)** Population quantification of impulsive lick rate across behavioral conditions. Each point represents an individual session (12 sessions across 5 animals), and gray lines connect trials from the same session. Box plots indicate median and interquartile range. Focal cooling significantly reduced the baseline impulsive lick rate compared to control conditions (one-way ANOVA, F=4.78, p=0.036). Recovery trials showed partial return toward baseline levels.


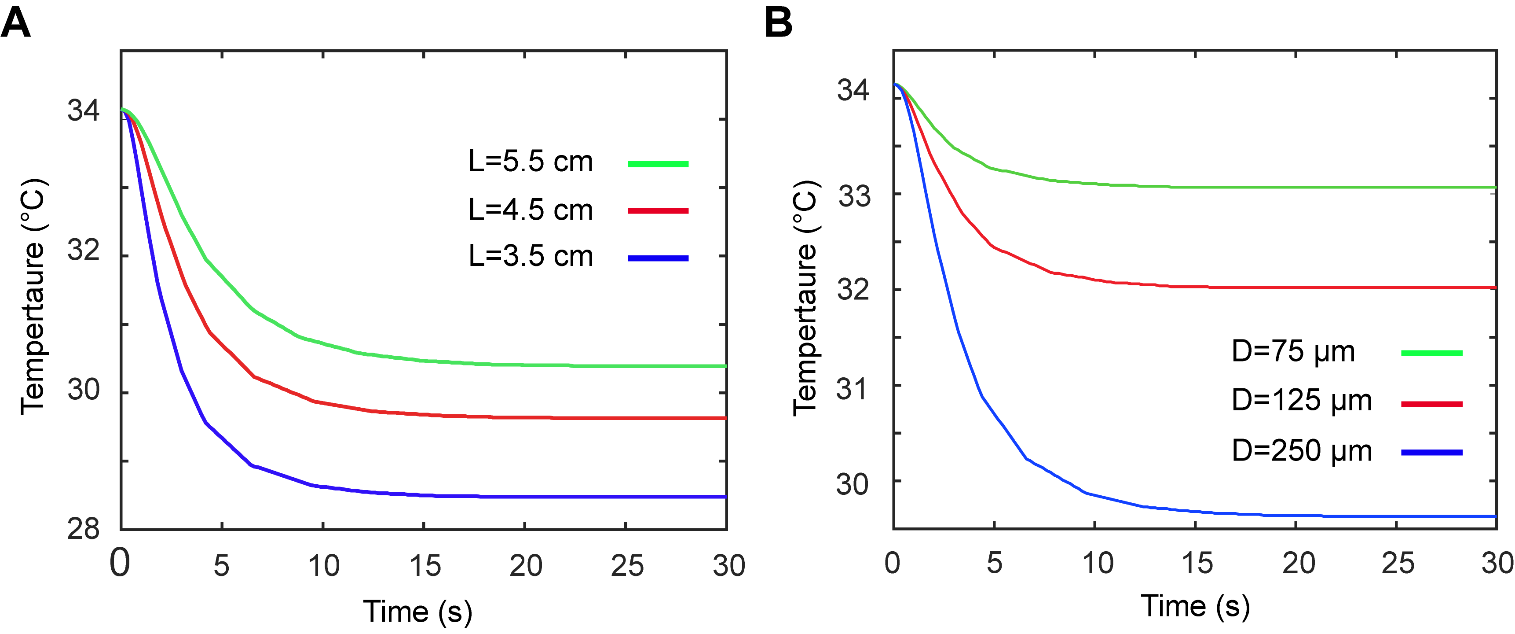


**Fig. S6.** **Finite element simulation results for focal cooling of a spot on the slice in a recording chamber**

**(A)** The temperature at the tip of the silver probe as a function of time for the probe with 250µm diameter and various lengths.

**(B)** The temperature at the tip of the probe as function of time for a probe with 4.5cm length and various diameters. Based on these results, we built a 250 µm diameter probe with the length of 3.5cm for the experiments.


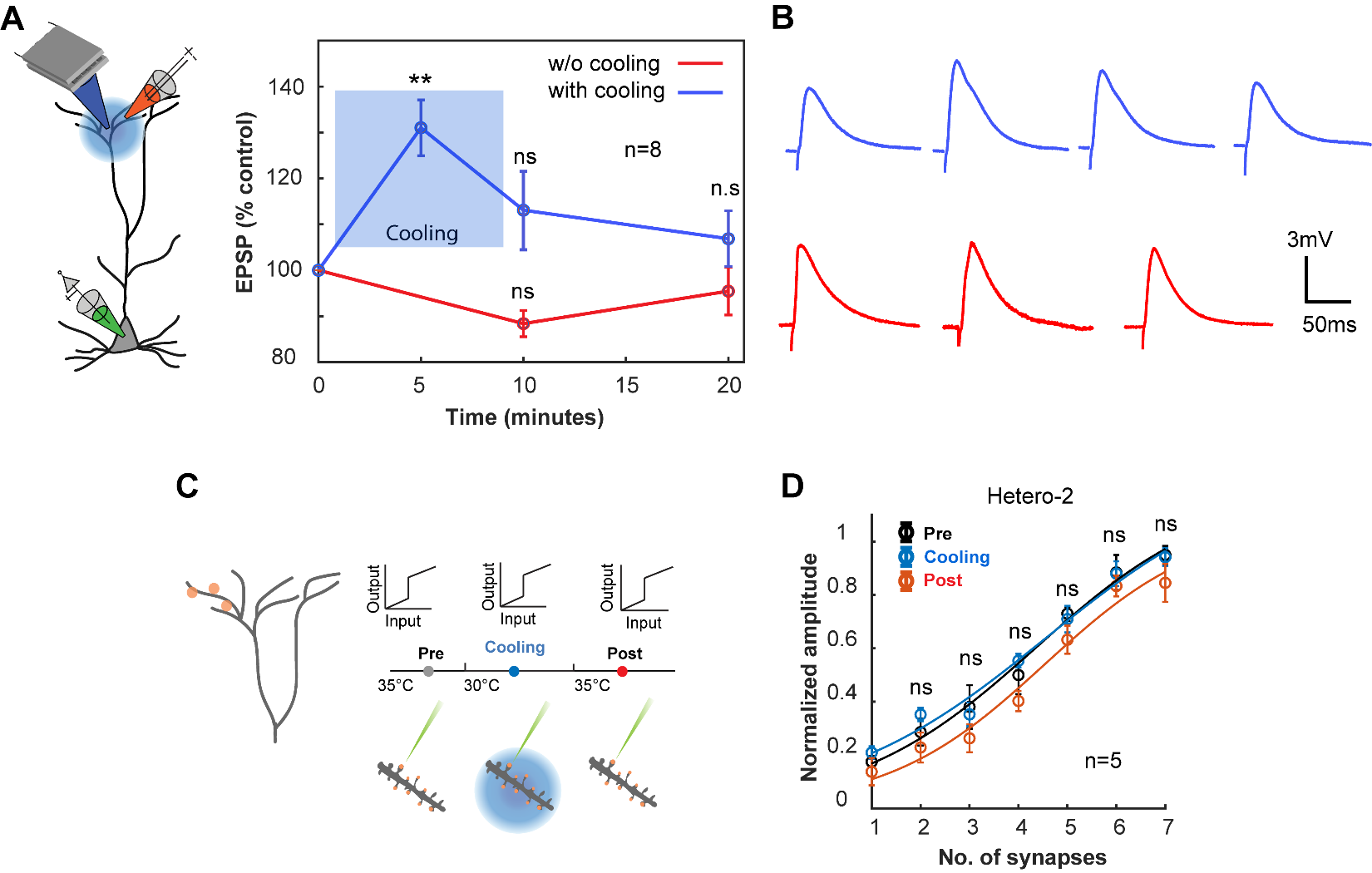


**Fig. S7. Cooling reversibly enhances the excitability of the tuft dendrite in a Kv4.2-dependent manner**

**(A)** Field-evoked EPSP amplitude measured at different times in the presence and absence of mild focal temperature modulation. All the readouts at 1 Hz. The readouts at time t=0, 10, and 20 all are performed at 35°C, the readout during the cooling phase (blue block) is performed at 29°C (Repeated-Measures ANOVA test, n=8, **p<0.01, ns, p>0.05).

**(B)** Example EPSPs shown at different times for cooling and control (no-cooling) experiments.

**(C)** Schematic of the experimental protocol for probing the temperature dependence of excitability via a readout of nonlinear input-output transformations across the tuft dendrite in the absence of plasticity induction. Two-photon glutamate uncaging probes nonlinear input-output characteristics before (control, grey), during (blue), and after (post, red) cooling.

**(D)** Normalized dendritic input-output response measured at the tuft dendrites before (35^°^C), during (29^°^C), and after (35^°^C) focal cooling in the presence of Kv4.2 blocker (heteropodatoxin-2) (Repeated-Measures ANOVA test, n=5, ns, p>0.05).


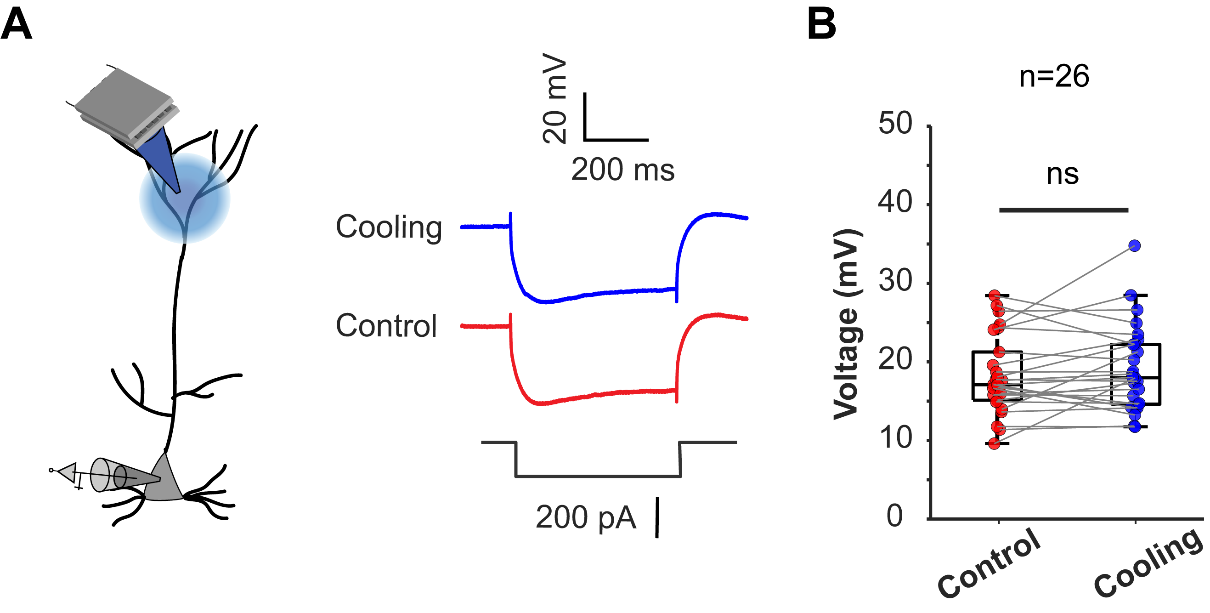


**Fig. S8. Somatic voltage as a function of tuft dendrite cooling**

**(A)** Schematic of the somatic patch clamp recording while cooling the tuft dendrite (left). The voltage response resulted from a subthreshold current injected in the soma (gray).

**(B)** Comparison of steady-state somatic voltage responses to somatic current injection at control temperature (red) and during tuft dendrite cooling (blue). Each pair of connected dots represents measurements from the same cell (n = 26 neurons). Recordings were made at the soma. No significant difference was detected between conditions (*Repeated-Measures ANOVA*, p = 0.267).


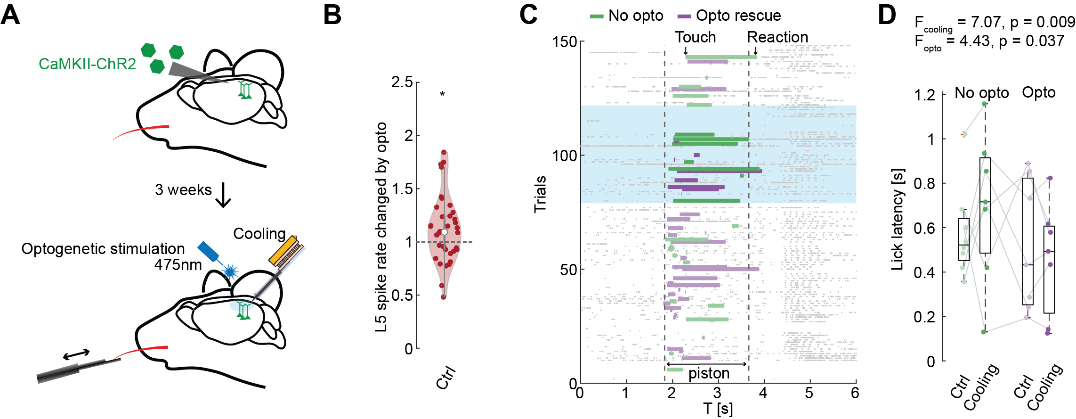


**Fig. S9. Cooling-induced behavioral impairment is rescued by optogenetic activation of the superficial layers**

**(A)** The experimental setup with simultaneous cooling and optogenetics in behaving mice. **(B)** The photostimulation induced change in spike rate of L5 single units under control condition (n = 39 L5 single units from 5 sessions, 2 animals, Wilcoxon signed rank test, * p < 0.05). **(C)** Lick raster with (purple) and without (green) optogenetic stimulation under control condition and cooling (blue box), the horizontal lines mark the time between the first whisker touch to the first lick after touch (lick latency). **(D)** The lick latency increases with cooling without optogenetic stimulation, but the increase is abolished with photostimulation (n = 8 sessions from 3 animals, two-way ANOVA).


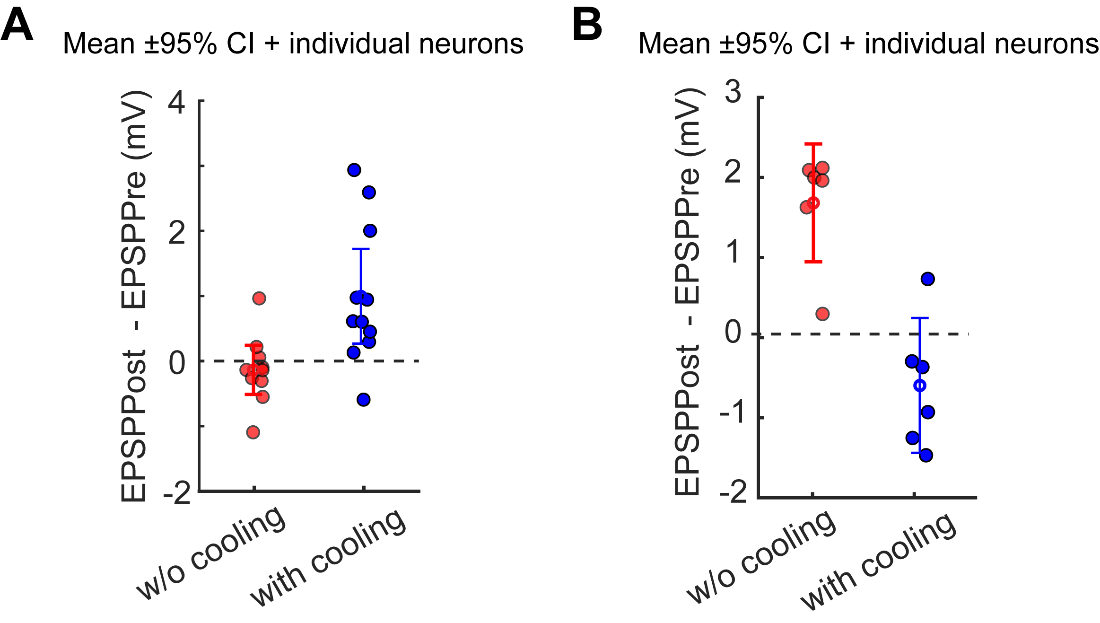


**Fig. S10. Variability and confidence intervals of compartment-specific dendritic plasticity**

**(A)** Distribution of plasticity magnitude in tuft dendrites following spike-timing–dependent plasticity (STDP) induction with and without mild focal cooling. Each dot represents one neuron and corresponds to the change in EPSP amplitude (ΔEPSP = EPSP_post_ − EPSP_pre_). Bars indicate the mean ± 95% confidence interval. Under mild focal cooling, ΔEPSP values are consistently positive and the confidence interval excludes zero, indicating robust potentiation across neurons.

**(B)** Same analysis for basal dendrites. In contrast to the tuft, STDP induction under mild focal cooling results in negative ΔEPSP values and a confidence interval that lies below zero, indicating depression across neurons. The dashed horizontal line denotes zero change in EPSP amplitude.


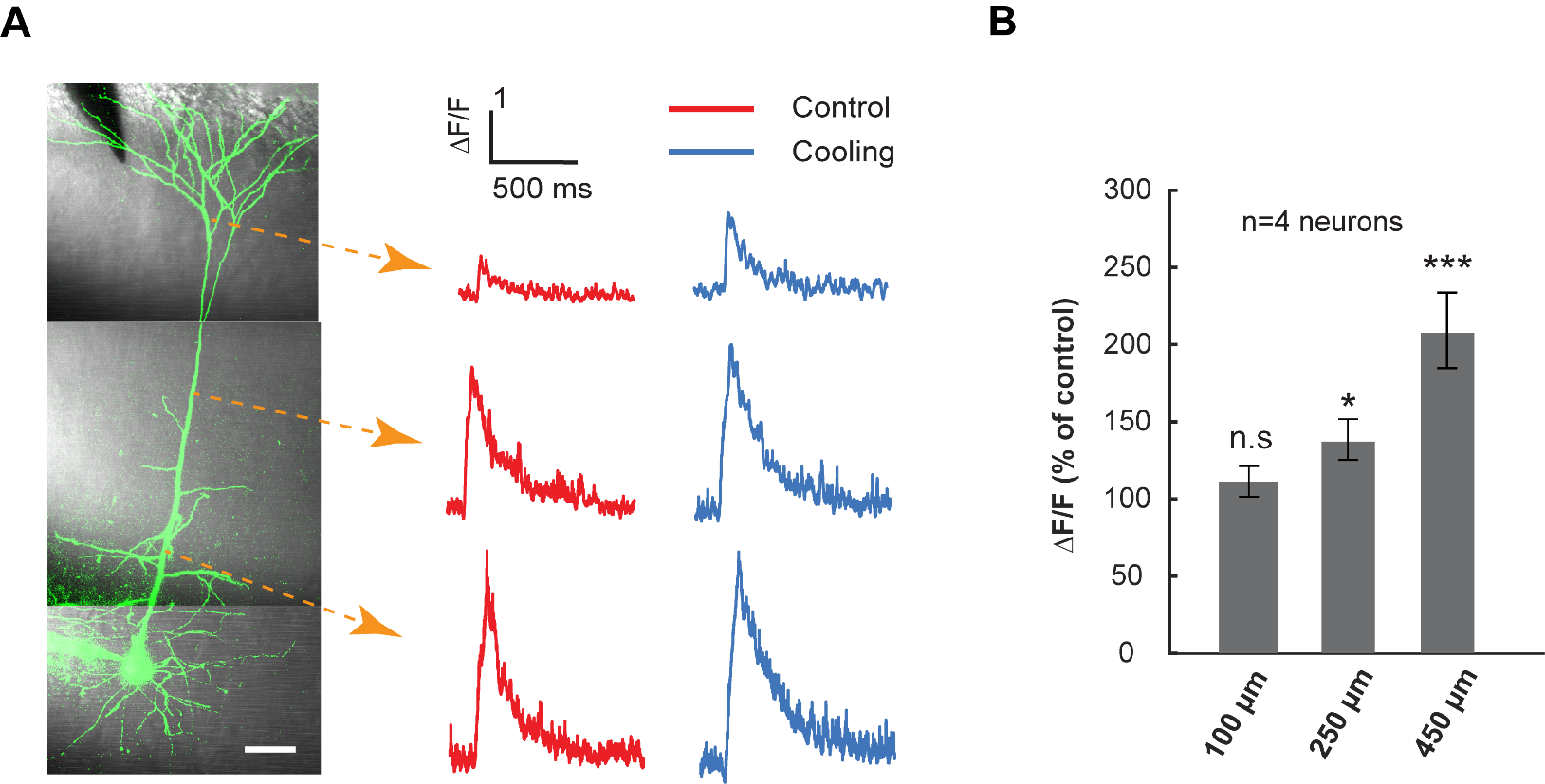


**Fig. S11. Cooling enhances dendritic calcium signals evoked by somatic bursts**

**(A)** Representative calcium transients (ΔF/F) recorded at different locations along the apical dendrite during a somatically generated burst of three action potentials, under control (red) and cooling (blue) conditions **(B)** Quantification of calcium responses at increasing dendritic distances (100, 250, and 450 μm) from the soma, normalized to control. Cooling significantly increased dendritic calcium signals, with stronger effects at distal sites (p=0.273 (100µm), p=0.045 (250µm), p=0.002 (450µm), One-way ANOVA test). Scale bar: 50µm.

***
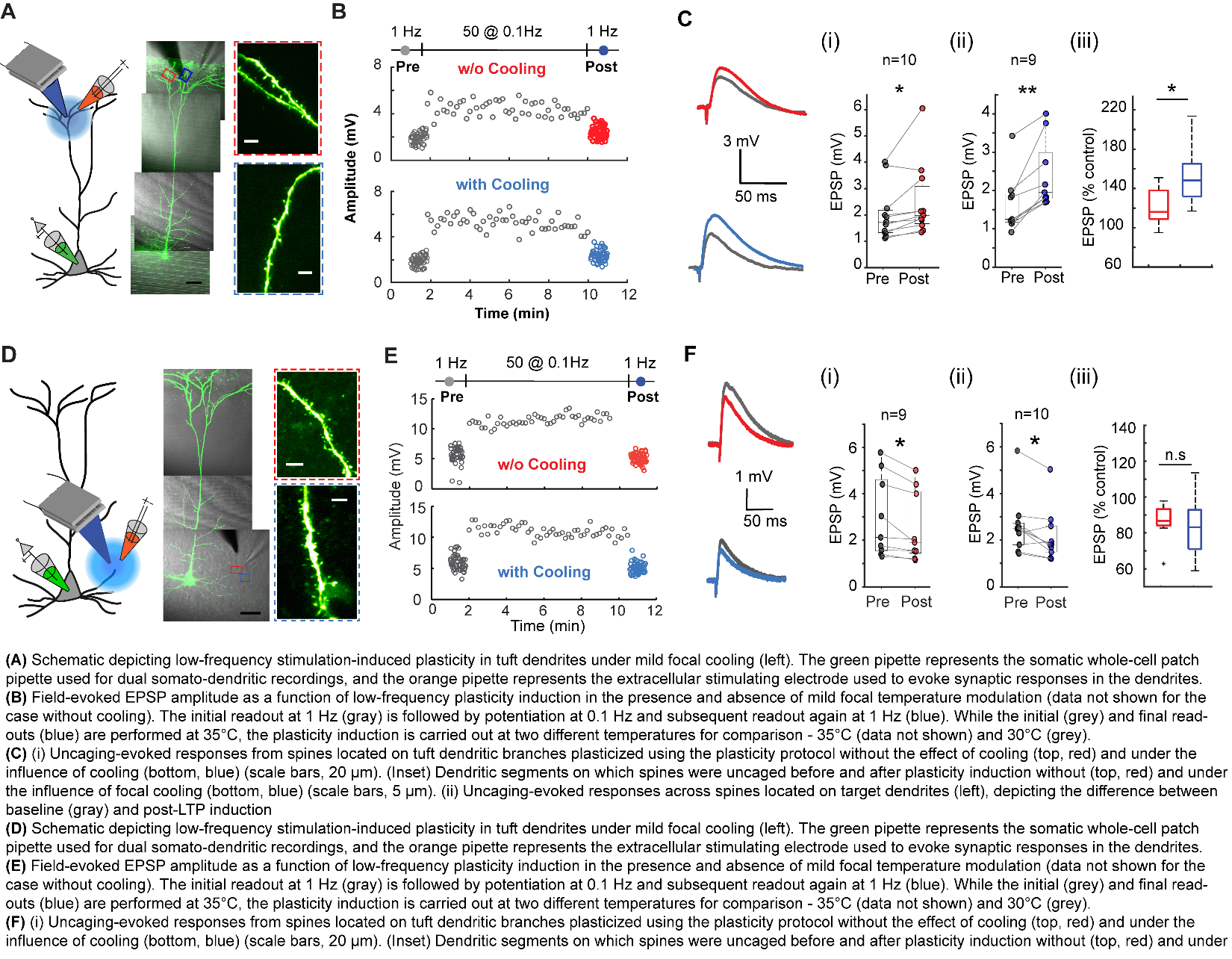
***

**Fig. S12. Mild focal cooling amplifies tuft dendritic plasticity and excitability**

**(A)** Schematic depicting low-frequency stimulation-induced plasticity in tuft dendrites under mild focal cooling (left). The green pipette represents the somatic whole-cell patch pipette used for dual somato-dendritic recordings, and the orange pipette represents the extracellular stimulating electrode used to evoke synaptic responses in the dendrites. An exemplar layer 5 pyramidal neuron loaded with Alexa 488 (100 μM) (scale bars, 50 µm). (Inset) Dendritic segments that underwent the plasticity protocol without (top, red) and under the influence of focal cooling (bottom, blue) (scale bars, 5 µm).

**(B)** Field-evoked EPSP amplitude as a function of low-frequency plasticity induction in the presence and absence of mild focal temperature modulation. The initial readout at 1 Hz (gray) is followed by potentiation at 0.1 Hz and subsequent readout again at 1 Hz (blue). While the initial (grey) and final readouts (blue) are performed at 35^°^C, the plasticity induction is carried out at two different temperatures for comparison - 35^°^C (data not shown) and 29^°^C (grey).

**(C)** Comparison between the percentage of increase (post vs. control) in the field-evoked EPSP amplitude while low-frequency potentiation is performed at different temperatures (protocol as described in (B). The induced plasticity is amplified under the influence of mild focal cooling ((i) pre vs post with cooling: 153%±11%, n=9, p=0.0039; (ii) pre vs post w/o cooling: 122%±5.6%, n=10, p=0.0068; Wilcoxon signed-rank test; (iii) overall EPSP enhancement with cooling compared to without cooling: p=0.027; Wilcoxon Mann-Whitney test). Error bars represent the standard error of the mean (SEM).

**(D)** Schematic depicting low-frequency stimulation-induced plasticity in basal dendrites under mild focal cooling (left). An exemplar layer 5 pyramidal neuron loaded with Alexa 488 (100 μM) (scale bars, 50 µm). (Inset) dendritic segments that underwent the plasticity protocol without (top, red) and under the influence of focal cooling (bottom, blue) (scale bars, 5 µm).

**(E)** Field-evoked EPSP amplitude as a function of low-frequency plasticity induction in the presence and absence of mild focal temperature modulation (data not shown for the case without cooling). The initial readout at 1 Hz (gray) is followed by potentiation at 0.1 Hz and subsequent readout again at 1 Hz (blue). While the initial (grey) and final readouts (blue) are performed at 35^°^C, the plasticity induction is carried out at two different temperatures for comparison - 35^°^C (data not shown) and 29^°^C (grey). Note the induced plasticity is not significantly changed under the influence of mild focal cooling.

**(F)** Comparison between the percentage of increase (post vs. control) in the field-evoked EPSP amplitude while low-frequency potentiation is performed at different temperatures. Results of field stimulation show a significant reduction in the EPSP amplitude after LTP ((i) pre vs post with cooling: p=0.015, n=10 neurons; (ii) pre vs post w/o cooling: p=0.012, n =9 neurons; Wilcoxon signed-rank test; (iii) overall EPSP enhancement with cooling compared to without cooling: p=0.23; Wilcoxon Mann-Whitney test). Error bars represent the standard error of the mean (SEM).


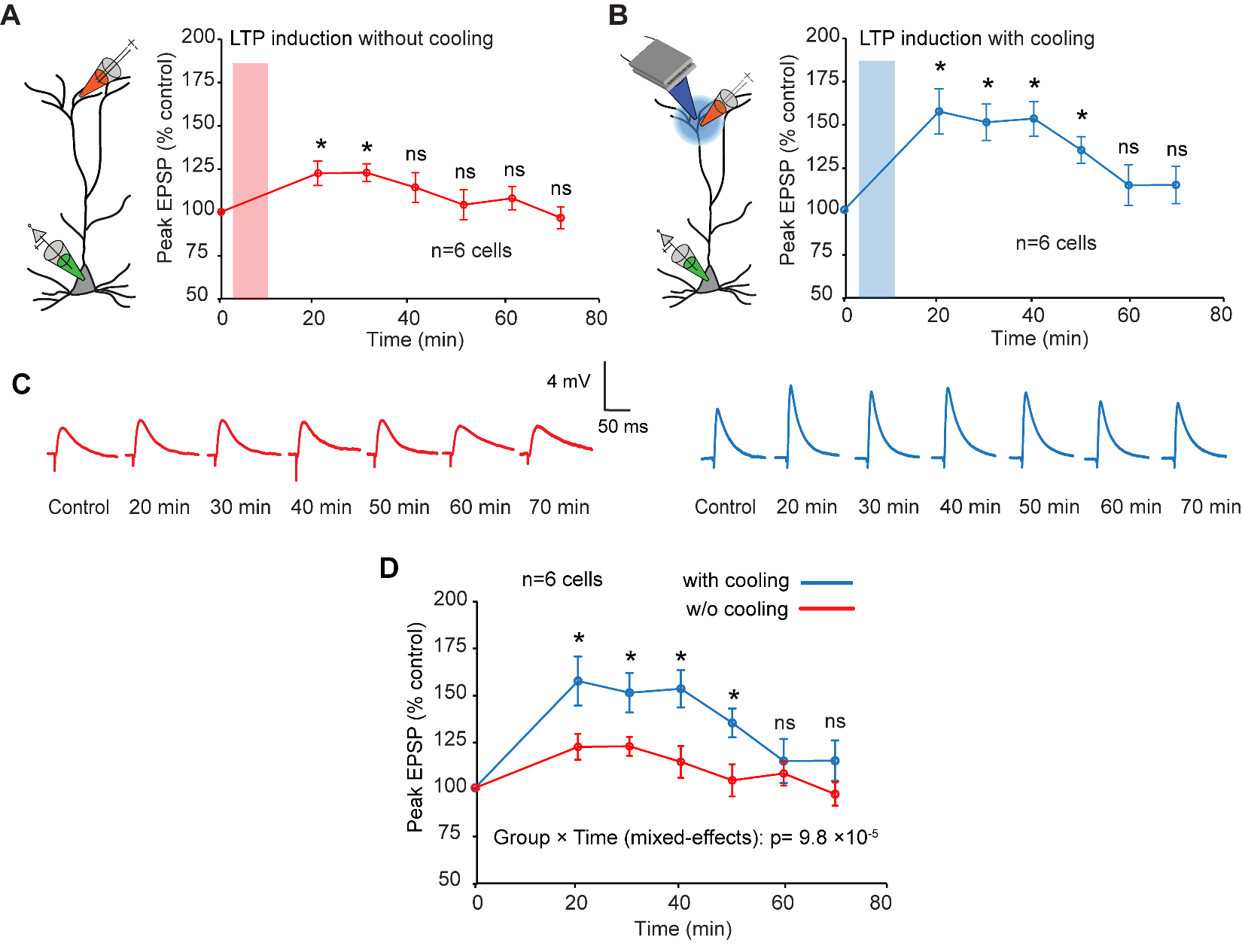


**Fig. S13. Time course of the low frequency induced synaptic plasticity at the tuft dendrite**

Field-evoked EPSP amplitude as a function of low-frequency plasticity induction in the presence and absence of mild focal temperature modulation. The initial readout at 1 Hz is followed by potentiation at 0.1 Hz and subsequent readouts at 1 Hz and at different times after the low-frequency induction. While the initial (pre) and post-induction readouts are performed at 35°C, the plasticity induction is carried out either at 35°C (for the “without cooling” experiment) (A) or 29°C for the experiment with cooling (B).

**(A)** Percentage increase (post-LTP vs. baseline control) of field-evoked EPSP amplitude recorded every 10 minutes after the LTP induction at physiological temperature (Wilcoxon signed rank test, n=6 neurons, *p<0.05). (Error bars represent the standard error of the mean (SEM)). **(B)** The same results as in A in the presence of cooling while LTP induction is performed. **(C)** Example EPSP at different points of time for the plasticity induction in the absence (red) or presence of (blue) of cooling. **(D)** Comparison between the time course of the plasticity effect for the induction at 35°C (red) and 29°C (blue) (Wilcoxon Mann-Whitney test, n=6 neurons, *p<0.05). Direct comparison of the time course between cooling and no-cooling conditions revealed a significant Group × Time effect (mixed Group × Time ANOVA implemented as a linear mixed-effects model, p = 9.8 × 10⁻⁵). Error bars represent the standard error of the mean (SEM).

*
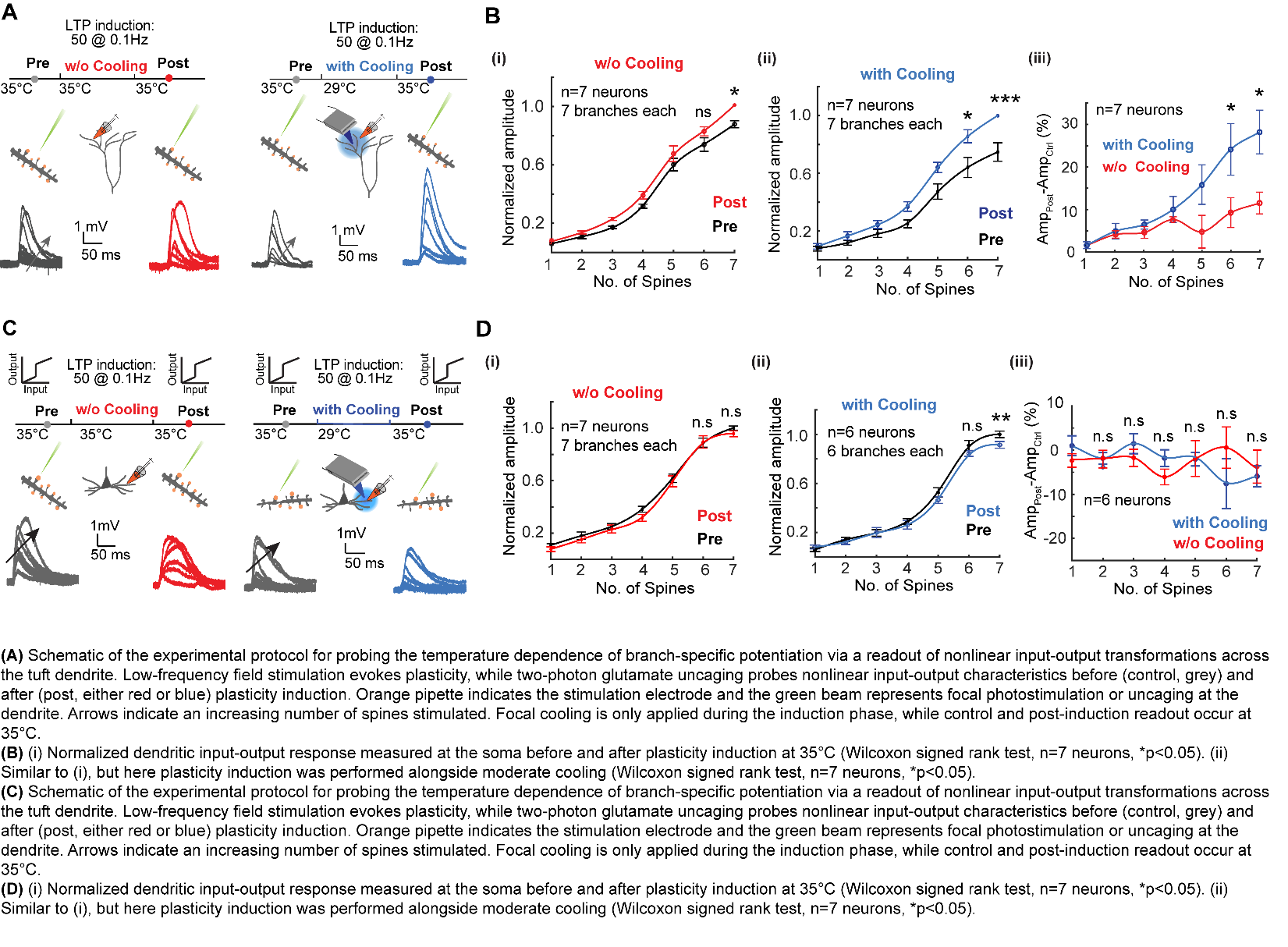
*

**Fig. S14. Cooling enhanced low frequency plasticity in tuft/basal dendrites as a function of number of evoked spines**

**(A)** Schematic of the experimental protocol for probing the temperature dependence of branch-specific potentiation via a readout of nonlinear input-output transformations across the tuft dendrites. Low-frequency field stimulation is used to evoke plasticity, while two-photon glutamate uncaging probes nonlinear input-output characteristics before (control, grey) and after (post, either red or blue) plasticity induction. The green beam represents focal photostimulation or uncaging at the dendrite. Arrows indicate an increasing number of spines stimulated. Focal cooling is only applied during the induction phase, while control and post-induction readout occur at 35^°^C.

**(B) (i)** Normalized dendritic input-output response measured at the soma before and after plasticity induction at tuft dendrite at 35^°^C (Wilcoxon signed rank test, n=7 neurons, *p<0.05). **(ii)** Similar to (i), but here plasticity induction was performed alongside moderate cooling (Wilcoxon signed rank test, n=7 neurons, *p<0.05). **(iii)** Comparison showing the percentage of amplitude increase for plasticity induced with focal cooling and without focal cooling (Wilcoxon Mann Whitney test, n=7 neurons, *p<0.05). Error bars represent the standard error of the mean (SEM).

**(C)** Schematic of the experimental protocol for probing the temperature dependence of branch-specific potentiation via a readout of nonlinear input-output transformations across the basal dendrites. Low-frequency field stimulation is used to evoke plasticity, while two-photon glutamate uncaging probes nonlinear input-output characteristics before (control, grey) and after (post, either red or blue) plasticity induction. The green beam represents focal photostimulation or uncaging at the dendrite. Arrows indicate an increasing number of spines stimulated. Focal cooling is only applied during the induction phase, while control and post-induction readout occur at 35^°^C.

**(F)** **(i)** Normalized dendritic input-output response measured at the soma before and after plasticity induction at basal dendrite at 35^°^C (Wilcoxon signed rank test, n=7 neurons, seven spines: p=0.3750, six spines: p=0.98125, five spines: p=0.3750, four spines: p=0.0313, three spines: p=0.2969, two spines: p=0.2969, one spine: p=0.1094). **(ii)** Similar to (i), but here plasticity induction was performed alongside moderate cooling (Wilcoxon signed rank test, n=6 neurons, seven spines: p=0.0303, six spines: p=0.4375, five spines: p=0.0313, four spines p=0.3125, three spines p=1.0, two spines p=0.3125, one spine p=0.3125). **(iii)** Comparison showing the percentage of amplitude increase for plasticity induced with focal cooling and without focal cooling (Wilcoxon Mann Whitney test, n=7 neurons, seven spines: p=0.5338, six spines: p=0.3660, five spines: p=0.7308, four spines: p=0.0734, three spines: p=0.5338, two spines: p=1.0, one spine: p=0.1014). Error bars represent the standard error of the mean (SEM).


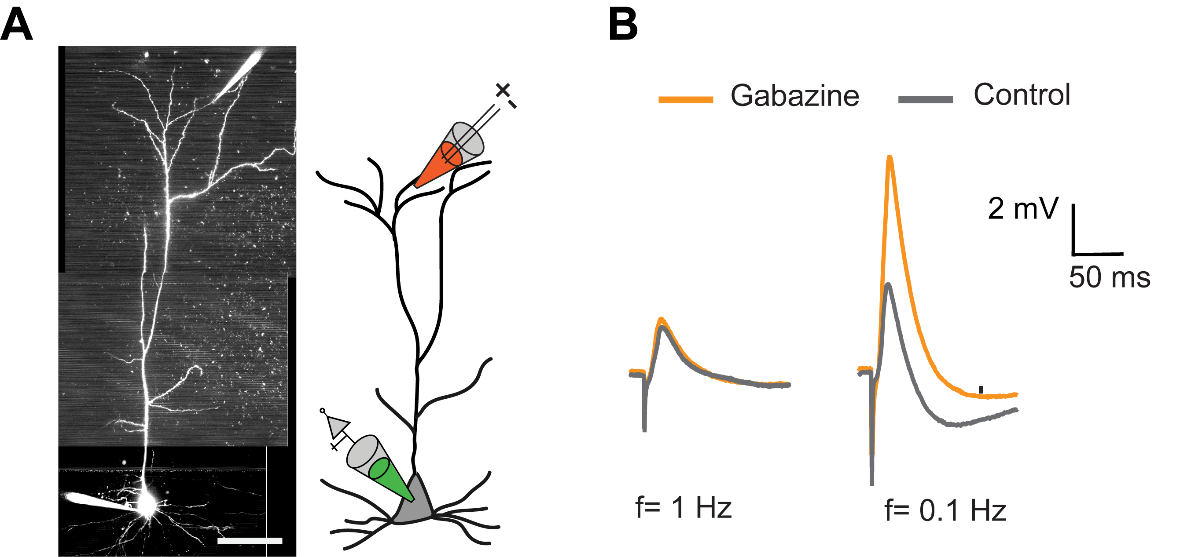


**Fig. S15. Gabazine blocks the inhibitions for low-frequency stimulation**

**(A)** Fluorescence image of a layer 5 pyramidal neuron loaded with Alexa 488 (100 μM) and the pipettes’ positions schematic. (**B)** Example EPSPs for the control experiment and after adding Gabazine are presented for different frequencies of field stimulation.

*
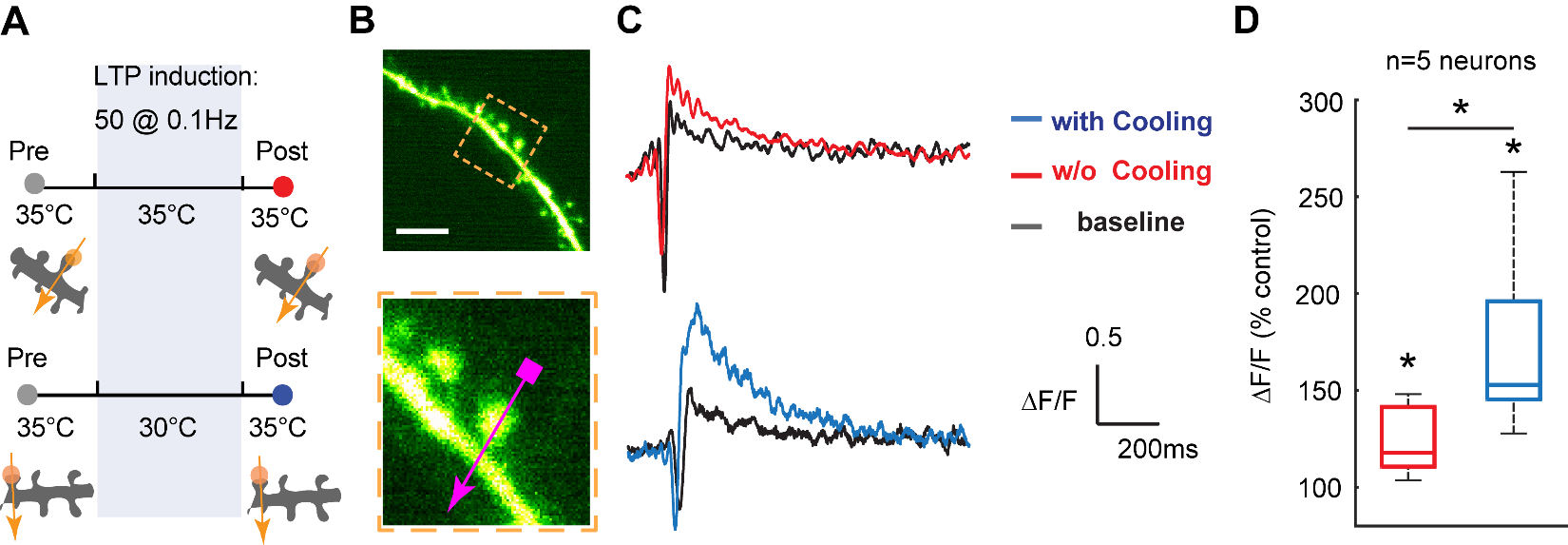
*

**Fig. S16. Mild focal cooling amplifies calcium transients in tuft dendrite**

**(A)** Schematic of the experimental protocol for probing the temperature dependence of low frequency induced plasticity at the tuft dendrite. Low-frequency field stimulation is used to induce plasticity. Two-photon calcium imaging triggered with spine uncaging is used to record the calcium transients before (control, grey) and after (post, either red or blue) plasticity induction. Focal cooling is only applied during the induction phase, while control and post-induction readout occur at 35°C. (**B)** Fluorescence image of the spine undergoing line scan calcium imaging (top) and the zoomed view (bottom). (Scale bar 5 μm). (**C)** Exemplar calcium transients showing the baseline (black) and post-LTP induction (red: 35°C; blue: 29°C; signifies the temperatures at which plasticity was carried out). (**D)** Percentage change (post-LTP vs. baseline control) of uncaging-evoked ΔF/F at the spine stimulated with mild focal cooling and without cooling (Wilcoxon signed rank test, n=5 neurons, *p<0.05) and comparison between the two (Wilcoxon Mann Whitney test, n=5 neurons, *p<0.05). Error bars represent the standard error of the mean (SEM).

***
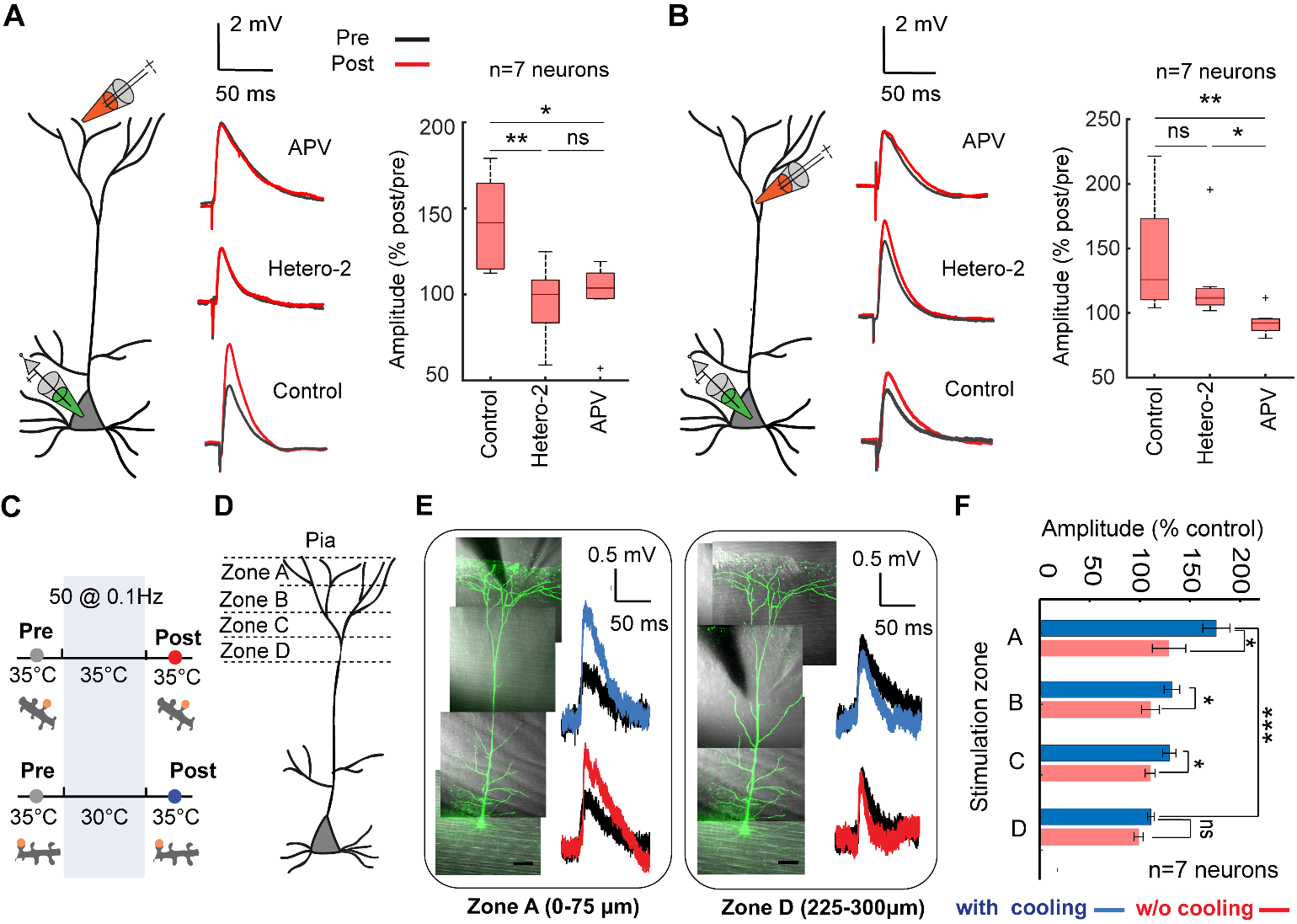
***

**Fig. S17. Gradient of plasticity along the apical dendrite**

**(A)** Experimental configuration. Field stimulation is used to evoke plasticity in the distal tuft dendrite (within 50 μm from pia) under the low frequency protocol. Example field-evoked EPSPs for pre (black) and post (red) stimulation for the control condition (bottom), after adding Heteropodatoxin-2 (Hetero-2, middle), and after adding APV (top). (Right) Comparison showing the percentage of EPSP increase (post vs. pre) with and without application of pharmacological blockers (Wilcoxon Mann Whitney test, n=7 neurons in each condition, Hetero-2 vs. control: p=0.046 and APV vs. control: p=0.0159). Error bars represent the standard error of the mean (SEM). **(B)** Similar experiment to that shown in (A) but performed in zone C at a different location of the tuft dendrite (within 200 μm from pia) (Wilcoxon Mann Whitney test, n= 7 neurons, Heter-2 vs. control: p= 0.9307, and APV vs. control: p=0.0159). Note the difference in both potassium channel dependence and amplification in plasticity in comparison to the distal tuft in (A). Error bars represent the standard error of the mean (SEM).

**(C)** Schematic of the experimental protocol for probing the gradient of the temperature dependence of low frequency induced plasticity along the tuft dendrite. Low-frequency field stimulation evokes plasticity, while two-photon glutamate uncaging is used to record the EPSP amplitude of control (grey) and after (post, either red or blue) plasticity induction. Focal cooling is only applied during the induction phase, while control and post-induction readout occur at 35^°^C. **(D)** Schematic depicting different distal tuft dendritic zones (zone A: 0-75 µm from pia, zone B: 75-150 µm from pia, zone C: 150-225 µm from pia, zone D:225-300 µm from pia). **(E)** Exemplar layer 5 pyramidal neurons (scale bars, 50 µm) shown for two different dendritic zones (zone A and zone D) that underwent field-evoked low-frequency plasticity. (Right) uncaging-evoked EPSPs depicting the difference between baseline (black) and post-LTP induction (red: 35^°^C; blue:29^°^C; signifies the temperatures at which plasticity was carried out). **(F)** Summary plot of the percentage of change in EPSP amplitude after LTP induction across different locations along the apical dendrite with and without mild focal cooling (Repeated-Measures ANOVA, n=7 neurons, *p<0.05, ***p<0.001). Error bars represent the standard error of the mean (SEM).


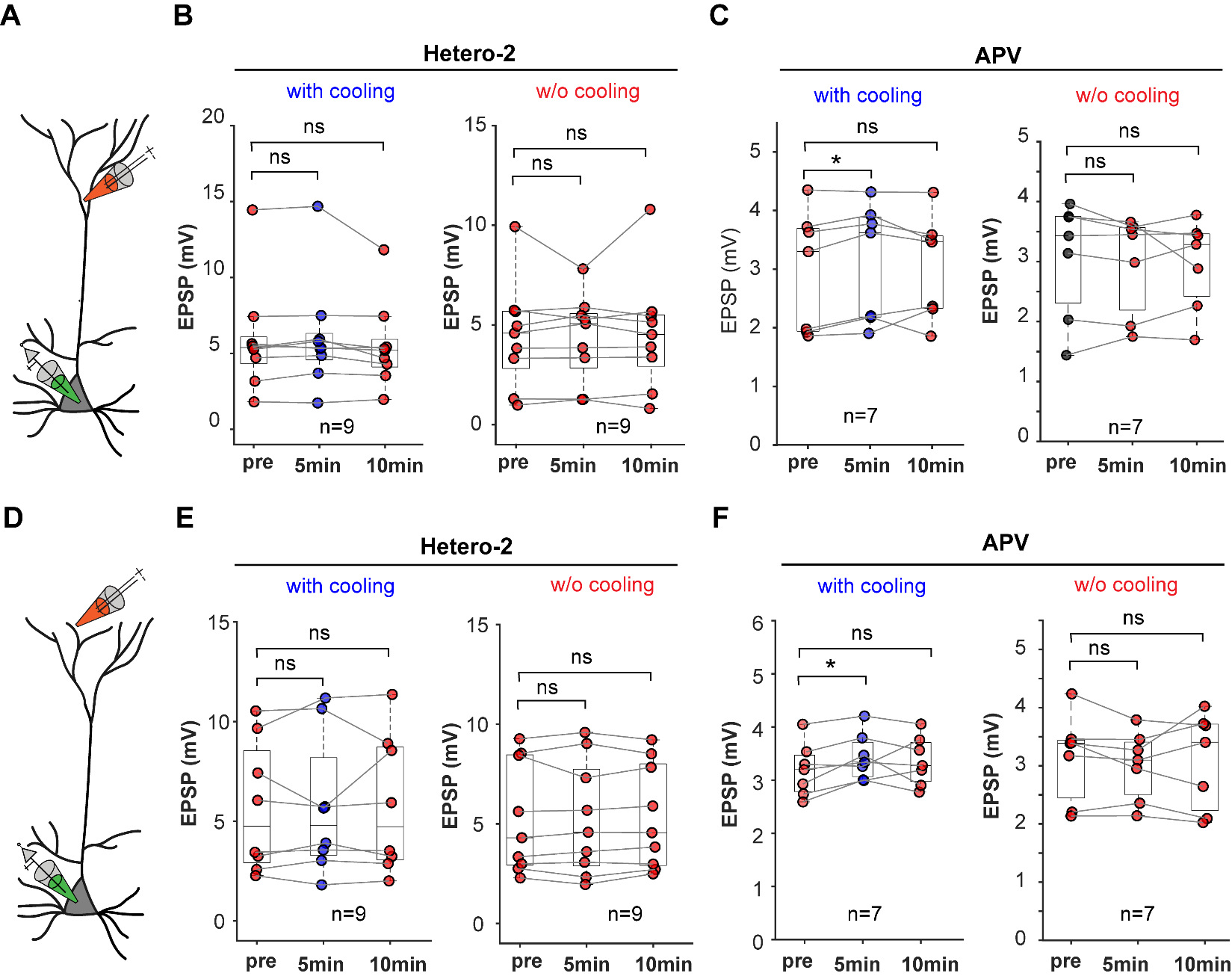


**Fig. S18. Time-matched controls confirm that pharmacological blockers affect plasticity induction, not baseline EPSPs**

**(A)** EPSP amplitudes recorded from initial segment of tuft dendrites (within 5-10um after bifurcation) in response to field-evoked low-frequency stimulation, with or without cooling and in the presence of Heteropodatoxin-2 (Kv4.2 channel blocker) (n=9 neurons, *p<0.05, ns, p>0.05; Repeated-Measures ANOVA) **(B)** or APV (NMDAR blocker) (n=7 neurons, *p<0.05, ns, p>0.05; Repeated-Measures ANOVA) **(C).** EPSPs were recorded at baseline (pre), 5 minutes, and 10 minutes after stimulation. In the absence of plasticity induction, EPSPs remained stable over time with or without cooling, indicating that the reduction in potentiation observed during cooling with Kv4.2 block reflects a disruption of plasticity mechanisms, not passive EPSP decay. **(D-F)** Same experimental layout as in (A-C) but from the stimulation of tertiary tuft segment. Regardless of the location along the tuft dendrite, blocking the Kv4.2 channels removes the excitability enhancement induced by cooling (n=9 neurons, *p<0.05, ns, p>0.05; Repeated-Measures ANOVA), however, NMDAR does not preclude the cooling-induced depolarization (n=7 neurons, *p<0.05, ns, p>0.05; Repeated-Measures ANOVA). Note: each line represents a single neuron. Cooling = blue; No cooling = red. Recordings were made from dendritic compartments using whole-cell patch clamp.

***
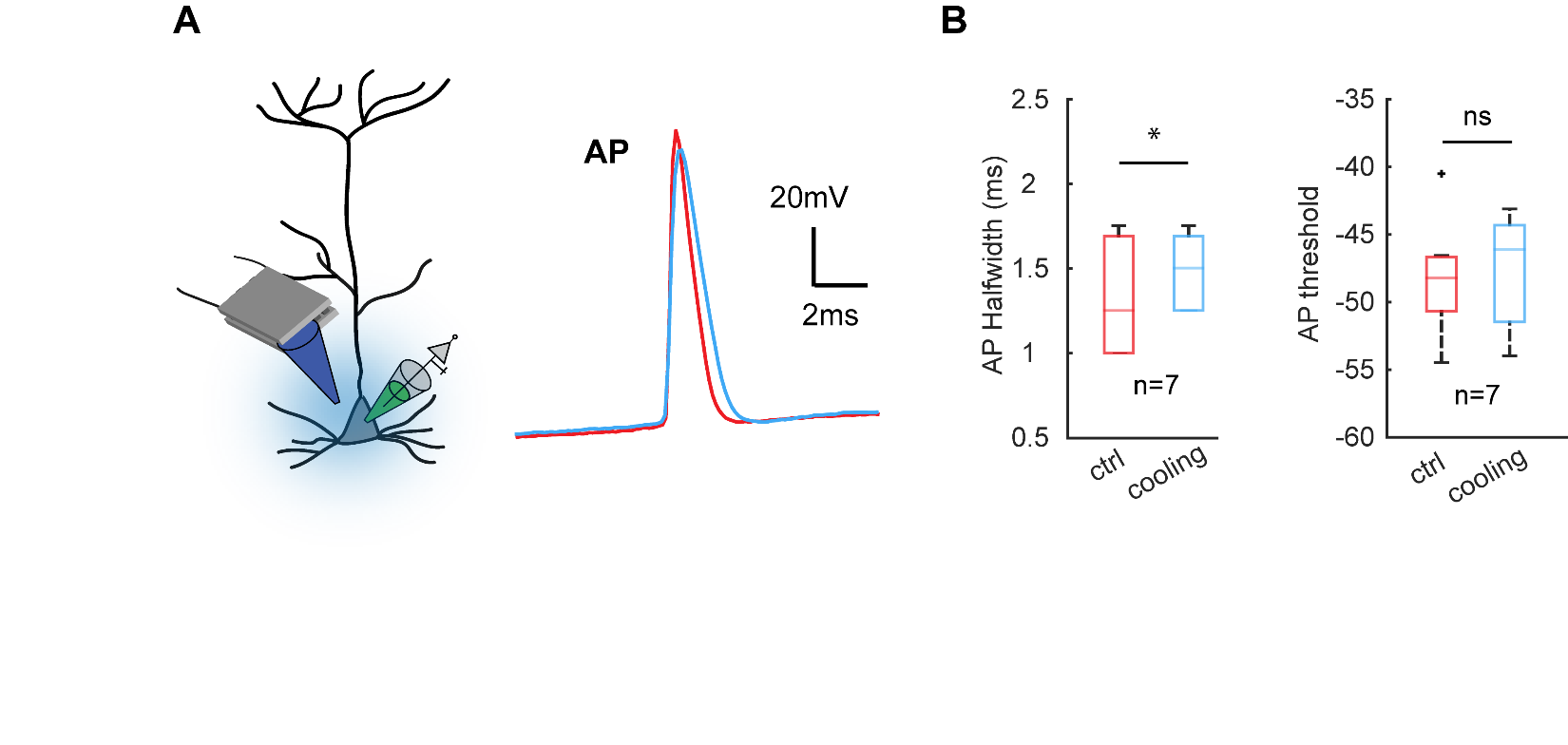
***

**Fig. S19. Cooling the soma increases the AP halfwidth without changing the threshold**

**(A)** Cell body is focally cooled using the cooling probe. Example action potential is plotted for cooling condition and control. **(B)** The half-width increases (Paired t-test, *p<0.05) by cooling and action potential threshold is not significantly affected (Paired t-test, ns, p>0.05). Box plots indicate the median and interquartile range; whiskers denote the data range.


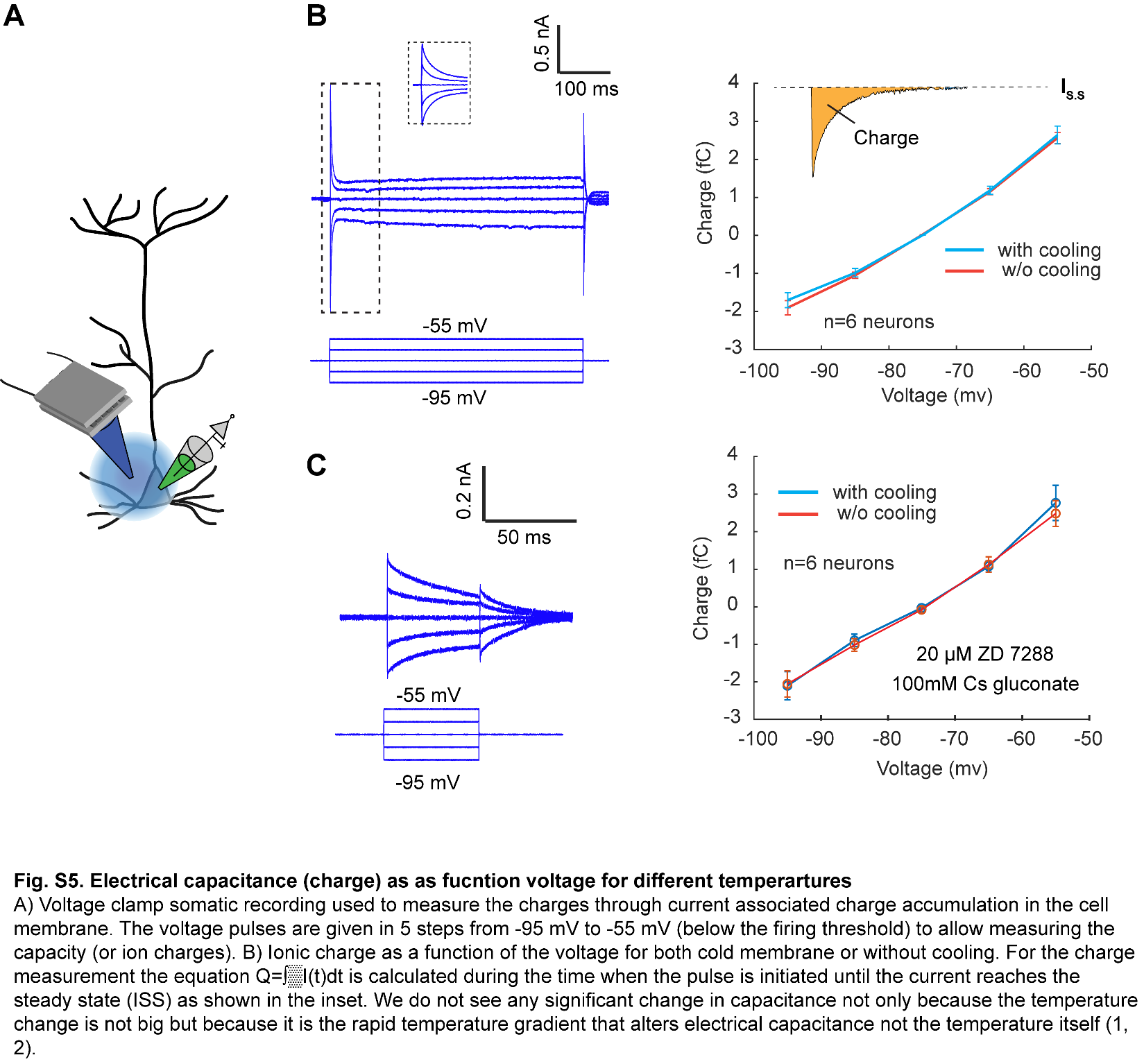


**Fig. S20. Electrical capacitance (charge) as a function of voltage for different temperatures**

***(A)*** *Voltage clamp somatic recording is used to measure the charges through current-associated charge accumulation in the cell membrane. The voltage pulses are given in 5 steps from -95 mV to -55 mV (below the firing threshold) to allow measuring the capacity (or ion charges).* ***(B)*** *Electric charge as a function of the voltage with and without cooling****.*** *For the charge measurement, the equation* $Q=\int I\left( t \right)dt$ *is calculated from the time the pulse is initiated until the current reaches the steady state (I_SS_), as shown in the inset. We do not observe any significant change in capacitance, possibly because the rapid temperature gradient alters electrical capacitance, not the temperature itself* (1, 2). ***(C)*** *The same experiments as performed in (B) in the presence of pharmacological blockers of HCN channels (20uM ZD7288 added to the ACSF bath) and rectifying potassium channels (100mM Cs Gluconate added to the internal solution).*

***
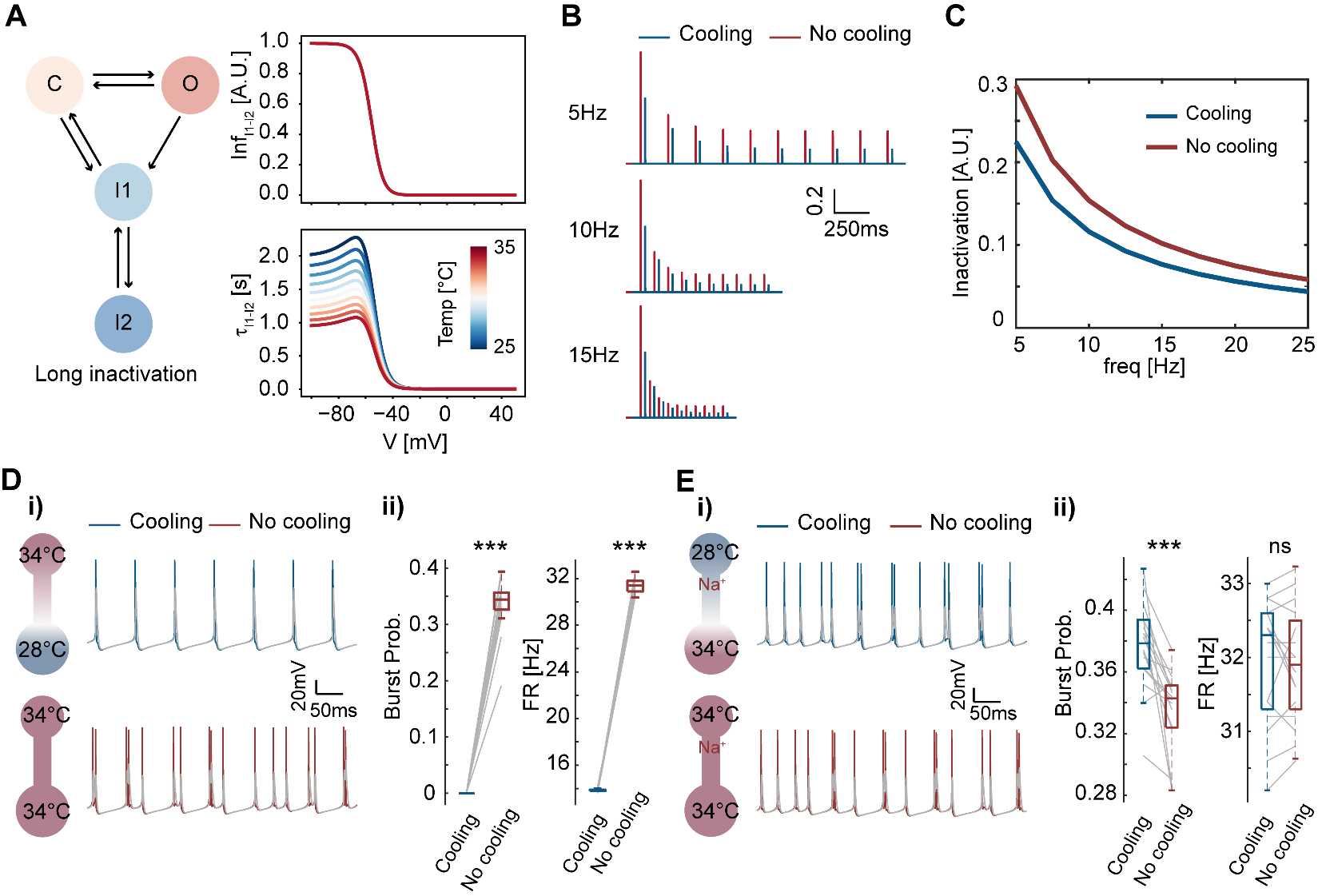
***

***Fig. S21. Slow recovery of Na^+^ channels from long-term inactivation resulted from cooling effects burstiness***

***(A)*** *(Left)* *Gating kinetic of the dendritic Na^+^ channels. (Right) Steady-state (top) and time constant (bottom) of the long-term inactivation gate (I_2_) as a function of temperature.* ***(B)*** *Dendritic Na^+^ channel gating under 10 short voltage pulses at different frequencies under mild cooling (blue, 28°C) and physiological temperature (red, 34°C).* ***(C)*** *Percentage of Na^+^ channels opening at the 10^th^ pulse compared to the 1^st^ pulse under cooling (blue) and physiological temperature (red).* ***(D)*** *i) Simulated spiking activities under somatic focal cooling (top) and physiological temperature (bottom), with gray traces indicating corresponding dendritic voltage. ii) Both burst probability and firing rate are reduced by somatic focal cooling (n = 20, Wilcoxon signed rank test, ***p < 0.001).* ***(E)*** Similar to D), but with focal cooling on apical dendrites, wherein dendritic Na^+^ channels exhibit no temperature modulation (n = 16, Wilcoxon signed rank test, ***p < 0.001).


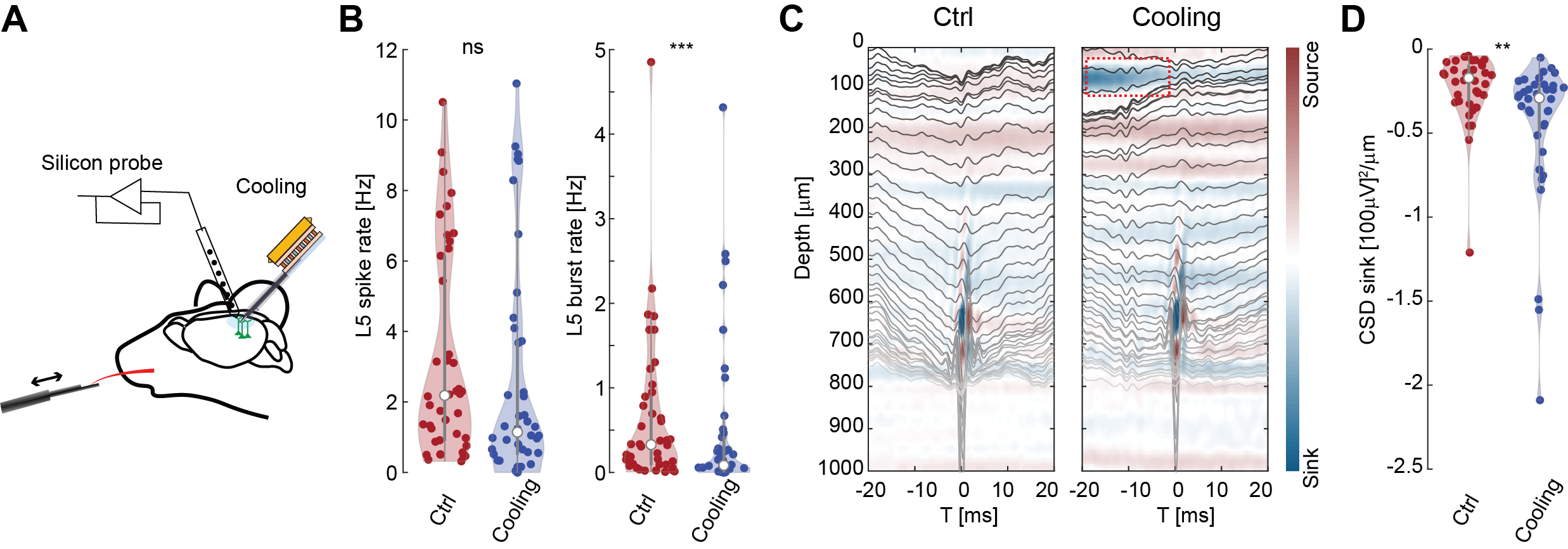


**Fig. S22. Surface focal cooling modulates burst but not spike rate of L5 neurons in vivo**

**(A)** The experimental setup with simultaneous cooling and silicon probe recording in behaving mice. **(B)** The spike rate and burst rate of L5 single units under control condition (red) and cooling (blue) (n = 42 L5 single units from 5 sessions, 2 animals, Wilcoxon signed rank test, *** p < 0.005). **(C)** The spike-triggered local field potential and current source density of an example L5 single unit under control condition and cooling. The red box highlights a prominent L1 sink proceeding the spikes under cooling. **(D)** Strength of the L1 current sink within 20ms before L5 spikes (n = 42 L5 single units from 5 sessions, 2 animals, Wilcoxon signed rank test, *** p < 0.01).

**Table S1: Parameters for the biophysical model**

| **Compartment** | **Parameter name** | **Value** |
| --- | --- | --- |
| global | e_na | 50 mV |
|  | e_k | -90 mV |
|  | e_ca | 140 mV |
|  | membrane time constant | 25 ms |
|  | Ca2+ diffuse gamma | 0.0006 |
|  | Ca2+ diffuse time constant | 35.7 ms |
| soma-AIS | g_leak | 0.03 mS/cm^2 |
|  | Cm | 0.75 µf/cm^2 |
|  | g_na | 3000 mS/cm^2 |
|  | g_k | 300 mS/cm^2 |
|  | area | 600 µm^2 |
| Distal apical | g_leak | 0.03 mS/cm^2 |
|  | Cm | 0.75 µf/cm^2 |
|  | g_nad | 20 mS/cm^2 |
|  | Im | 0.1 mS/cm^2 |
|  | g_ca | 0.3 mS/cm^2 |
|  | g_kca | 3 mS/cm^2 |
|  | distance to soma | 400 µm |
|  | rho (ratio of area to somatic area) | 20 |
|  | kappa (axial coupling resistance) | 5 Mohm |
| Proximal apical | g_leak | 0.03 mS/cm^2 |
|  | Cm | 0.75 µf/cm^2 |
|  | g_nad | 20 mS/cm^2 |
|  | g_k | 0.4 mS/cm^2 |
|  | distance to soma | 250 µm |
|  | rho (ratio of area to somatic area) | 15 |
|  | kappa (axial coupling resistance) | 5 Mohm |

**References**

1. Shapiro, M.G., Homma, K., Villarreal, S., Richter, C.-P., and Bezanilla, F. (2012). Infrared light excites cells by changing their electrical capacitance. Nat. Commun. *3*, 736. <https://doi.org/10.1038/ncomms1742>.

2. Liu, Q., Frerck, M.J., Holman, H.A., Jorgensen, E.M., and Rabbitt, R.D. (2014). Exciting cell membranes with a blustering heat shock. Biophys. J. *106*, 1570-1577. <https://doi.org/10.1016/j.bpj.2014.03.008>.
